# A simple two-state model interprets temporal modulations in eruptive activity and enhances multivolcano hazard quantification

**DOI:** 10.1126/sciadv.abq4415

**Published:** 2022-11-02

**Authors:** Jacopo Selva, Laura Sandri, Matteo Taroni, Roberto Sulpizio, Pablo Tierz, Antonio Costa

**Affiliations:** ^1^ Istituto Nazionale di Geofisica e Vulcanologia (INGV), Sezione di Bologna, Bologna, Italy.; ^2^ Istituto Nazionale di Geofisica e Vulcanologia (INGV), Sezione di Roma 1, Rome, Italy.; ^3^ Dipartimento di Scienze della Terra e Geoambientali, University of Bari, Bari, Italy.; ^4^ IGAG-CNR, Milano section, Milan, Italy.; ^5^ British Geological Survey (BGS), The Lyell Centre, Edinburgh, UK.

## Abstract

Volcanic activity typically switches between high-activity states with many eruptions and low-activity states with few or no eruptions. We present a simple two-regime physics-informed statistical model that allows interpreting temporal modulations in eruptive activity. The model enhances comprehension and comparison of different volcanic systems and enables homogeneous integration into multivolcano hazard assessments that account for potential changes in volcanic regimes. The model satisfactorily fits the eruptive history of the three active volcanoes in the Neapolitan area, Italy (Mt. Vesuvius, Campi Flegrei, and Ischia) which encompass a wide range of volcanic behaviors. We find that these volcanoes have appreciably different processes for triggering and ending high-activity periods connected to different dominant volcanic processes controlling their eruptive activity, with different characteristic times and activity rates (expressed as number of eruptions per time interval). Presently, all three volcanoes are judged to be in a low-activity state, with decreasing probability of eruptions for Mt. Vesuvius, Ischia, and Campi Flegrei, respectively.

## Teaser

A simple two-state statistical model can describe the temporal modulations in eruptive activity of Neapolitan volcanoes.

## INTRODUCTION

Volcanic processes occur over a very wide range of spatial, temporal, and energy scales, leading to an extraordinary variability in behaviors [e.g., (*1*, *2*)]. Eruptive histories, which are typically reconstructed from geological records and historical chronicles, widely vary among volcanoes, covering the whole range between continuous activity with sporadic larger eruptions and sporadic (large) eruptions after centuries-long resting periods.

Frequently erupting volcanoes are often characterized by flares in their activity or more complex patterns such as open-conduit periods with a relatively high frequency of eruptions due to the presence of eruptible magma in shallow reservoirs [e.g., (*3*)] and closed-conduit regimes characterized by the lack of eruptible magma and plugged conduits [e.g., (*4*, *5*)]. Less frequently erupting volcanoes with a relatively long reconstructed eruptive history often show substantial changes in their long-term activity as well, with modulations in terms of activity rate and/or eruptive style, which include the existence of specific trends in activity (both increasing and decreasing) or cyclical behaviors [e.g., (*6*)].

These complex temporal behaviors have been modeled using different statistical approaches [for a review, see (*7*)] that range from constant-rate homogeneous Poisson models to different flavors of renewal processes [e.g., Brownian passage-time, Weibull, and Gamma distributions], including size/time predictable and non-homogeneous Poisson models [e.g., (*6*, *8*–*12*)]. Given that several volcanoes show the existence of episodes characterized by a series of events followed by long repose times, several authors suggested a specific class of models that assumes that volcanoes randomly oscillate through time between discrete regimes, each characterized by a specific stochastic behavior with random duration [e.g., (*4*, *7*, *12*–*18*)].

Reference models applicable to a wide range of volcanoes do not exist. Hence, different choices are made for different volcanoes, depending on existing specific conceptual models and/or based on working hypotheses derived from data. This complicates the comparison among volcanoes, because existing estimates are heterogeneous and linked to different assumptions, and prevents an effective comparative discussion of the physical processes generating the time series. The possible ground for a common reference model can be found in volcanic regimes (*7*). At most active volcanoes, magma is not always available in shallow reservoirs and, even when this magma is available, the conditions for eruptions are not always there [e.g., (*19*–*21*)]. Conditions for magma rising and eruption are determined by the complex interaction of magma overpressure and local stress conditions [e.g., (*3*, *22*, *23*)]. The eruptions themselves may induce structural weakening of the volcanic system and, on the other side, may potentially trigger further magma rise from depth by increasing the pressure gradient [e.g., (*12*, *24*)]. The occurrence of one eruption is always a rupture of equilibrium between magmatic pressures and confining forces, which usually takes time to restore. This may determine, in the short term after an eruption, the right conditions for new eruptions. However, these conditions may vanish through time as the equilibrium is restored, due to a variety of processes such as closure of conduits, cooling of magma, magma crystallization, degassing, and limited injection of magma batches into the crust or in the magma chamber(s) (*25*–*27*). This implies that, in many volcanoes, (at least) two states should exist, with periods of high and low activity, and that the switch between these two states is not an independent random process, but a process that can be characterized by the observable eruptive history of a volcano. Thus, a model homogeneously quantifying, for different volcanoes, the characteristics of the (two) different states and the timing for restoring equilibrium may provide important clues about the main processes leading to eruptions at each volcano, as the processes eventually leading to an eruption have different time scales.

Understanding and forecasting eruptive activity is also critical, as volcanoes may pose important risks to humans and built environments, especially in densely inhabited areas, such as the metropolitan area of Naples, Italy [e.g., (*28*, *29*)]. Volcanoes can generate a large number of possible hazardous phenomena, ranging from tephra fallout to pyroclastic flows, lava, gasses, tsunami, and many others [e.g., (*30*)]. In some cases, volcanoes are clustered in space so that any single target may be affected by multiple volcanoes [e.g., (*1*)]. For long-term hazard quantification purposes, with inference time windows of tens of years to centuries or more (*31*), the difficulty in defining a consistent temporal behavior for eruptions in many cases has pushed research toward the quantification of the hazard conditional upon the occurrence of one eruption (*32*–*36*). Very different techniques have been developed to quantify the probability of eruption in a given time window, ranging from counts of months without unrest (*37*, *38*) to the application of different statistical distributions [e.g., (*9*, *12*, *18*, *39*)], to the development of more complex self-exciting nonhomogeneous processes (*40*, *41*). Often, such methods concentrate on the next eruption, neglecting the possibility of multiple eruptions or of changes in the eruptive regime. The lack of reference models and homogeneous estimations of the probability of eruption also complicates the formulation of multivolcano hazard quantifications (*28*, *29*, *42*), which are fundamental in areas potentially affected by multiple volcanoes, and prevents the inclusion of volcanic hazard into multihazard risk studies [e.g., (*43*–*45*)].

Here, we develop a simple two-state statistical model in which the transition between low- and high-activity regimes is linked to the physics of the system as recorded in the eruption catalogs. The model, which is controlled by only three parameters, is fitted to the three active volcanoes in the Neapolitan area, Italy, which are exemplificative of three very different styles of volcanism: Mt. Vesuvius, a central strato-volcano with a clear open-/closed-conduit history (*4*, *37*, *46*, *47*); Campi Flegrei, a large silicic caldera formed by at least two major collapses (*48*–*50*); and Ischia, which is the emerged part of a volcanic field rising more than 1000 m above the sea floor and morphologically dominated by one of the largest intracalderic asymmetric resurgence (*51*–*53*). The obtained models are first compared with the known eruptive histories. Then, they are used to compare the eruptive conditions at the three volcanoes, and to produce a unified picture of the overall volcanic hazard with a homogeneous quantification of the probability of eruption.

## RESULTS

### Model development

**Fig. 1.**
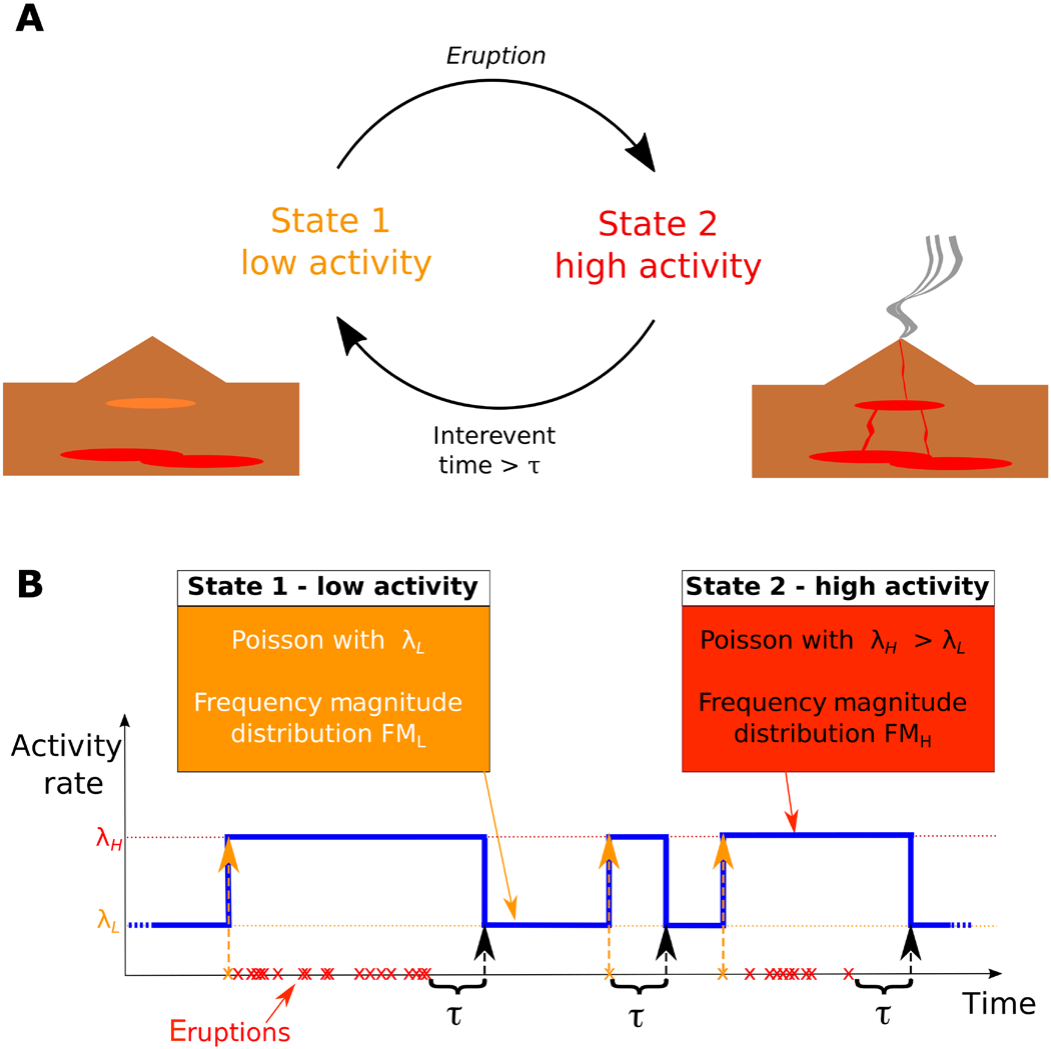
Conceptual model. (**A**) Reference conceptual model for volcanoes, switching between low- and high-activity states based on eruption history. (**B**) Simplest implementation of the conceptual model, with both states characterized by homogeneous Poisson processes with different annual rates and frequency-magnitude distributions: The high-activity states are triggered by the eruptions and are maintained until an interevent time larger than a threshold τ is observed.

The model is based on the hypothesis that volcanic regimes, which have their roots in the concept of alternating stress equilibrium and disequilibrium conditions occurring in volcanic systems [e.g., (*3*, *7*, *13*, *22*, *23*)], can be fully tracked through the eruptions. More specifically, when a volcano does not erupt for a sufficiently long period, its structural conditions may be assumed at equilibrium. The occurrence of an eruption, independently from its size, can lead the volcano into a state of high activity [e.g., (*3*, *12*, *22*–*24*)] or, if it was already in such a state, maintain it. In contrast, when the volcano does not erupt for a long-enough time, it falls back to a low-activity state of equilibrium. If the system has not been substantially modified by the eruptive activity, we may assume that the new equilibrium will be similar to the original state. This simple conceptual model is reported in Fig. 1A, and it is at the base of the formulation of the statistical model.

Even though more complex temporal behavior may also be foreseen [e.g., (*6*, *7*, *12*, *18*)], in its simplest implementation, we may assume that both high- and low-activity states are characterized by a homogeneous Poisson occurrence (Fig. 1B). In these conditions, when the volcano is in a low-activity state, it produces eruptions following a Poisson process with an annual rate (the average number of eruptions per year) λ*_L_*. When an eruption occurs, the system automatically switches to a high-activity state, in which eruptions are produced with a higher mean annual rate λ*_H_* (i.e., λ*_H_* > λ*_L_*). The volcano remains in high-activity state until a period without eruptions longer than a threshold interevent time τ occurs, so that the system falls back to a low-activity state. This model describing the temporal occurrence of eruptions is characterized by only three parameters: the activity rates in high- and low-activity states λ*_H_* and λ*_L_*, respectively, and the threshold interevent time τ. To complete the characterization of eruptive time series, specific frequency-magnitude distributions may be assumed to hold during the two states (see Materials and Methods).

This model configuration recalls a two-stage Poisson Markov process (*6*, *13*, *16*, *18*, *54*); however, here, the transition among the states is determined by the time history of eruptive sequences, and it has a clear physical interpretation in terms of the magmatic feeding system. In particular, the onset of high-activity states is controlled by the occurrence of an eruption, and any eruption deterministically pushes the system toward the high-activity regime. In this sense, our model recalls a self-exciting Cox-Hawkes process (*41*, *55*, *56*), in which each individual eruption can keep the system excited in a high-activity state. On the other hand, the transition to a low-activity state occurring after a long-enough repose time has some similarities with a Markov modulated Poisson process (*13*) in which the probability of transition changes as the time from the last eruption increases.

**Fig. 2.**
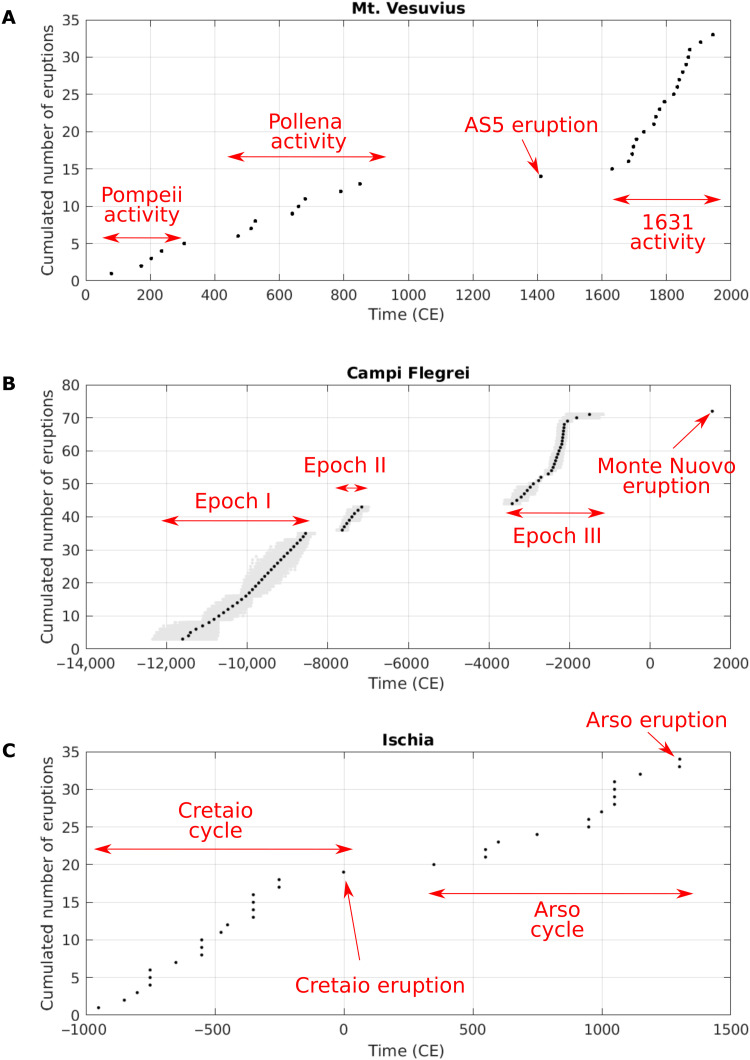
Low-/high-activity states. Identification of states of low/high activity in the eruptive history of (**A**) Mt. Vesuvius, (**B**) Campi Flegrei, and (**C**) Ischia. For Campi Flegrei, the gray areas indicate the uncertainty on the time of the eruption (see Materials and Methods).

All the three active Neapolitan volcanoes show clear clusters and isolated events in their eruptive history (Fig. 2). In particular, after its formation within the Somma caldera, Mt. Vesuvius has been characterized by three open-conduit phases, taking place after the three largest eruptions (Pompeii, Pollena, and 1631), and by one isolated event in the 15th century (AS5) (*46*). After the Neapolitan Yellow tuff caldera formation [ca. 14,000 years ago; (*57*)], Campi Flegrei experienced three epochs of activity (*49*), while its last eruption (AD 1538) occurred isolated, preceded and followed by centuries without activity. Also Ischia experienced at least two periods of high eruptive activity in the last 3000 years, culminated by the Cretaio and the Arso eruptions, respectively, which represent the largest explosive and effusive events in Ischia recent history (*51*–*53*). Such periods, defined on geological ground, are essentially confirmed by standard cluster analysis (see Materials and Methods). Notably, the stability of results with alternative clustering for both Campi Flegrei and Ischia is checked (see the Supplementary Materials).

### Parameter estimation

**Fig. 3.**
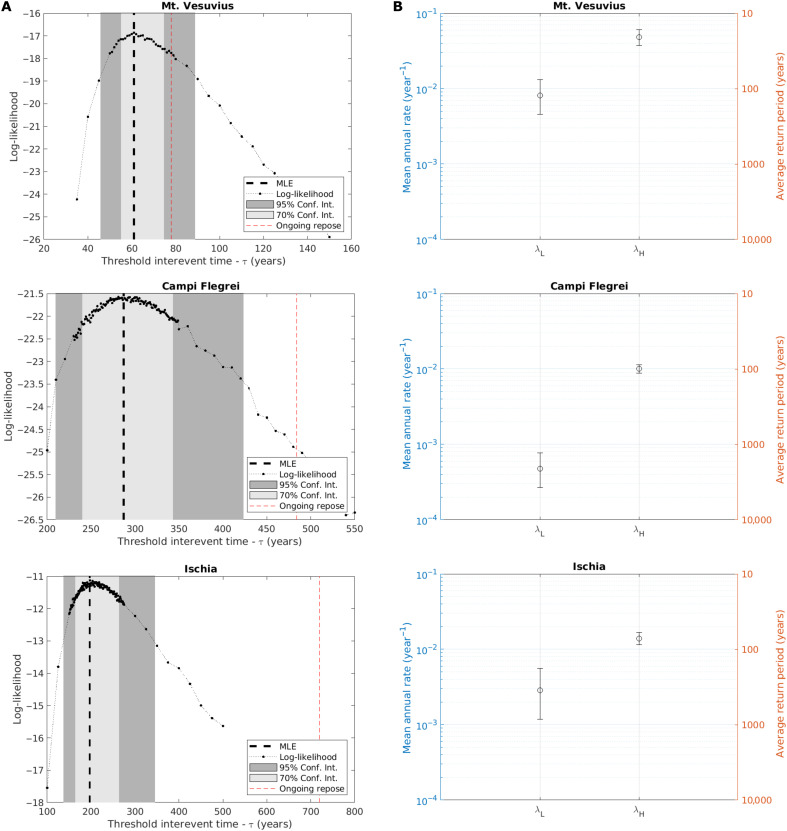
Parameter estimation. Parameter estimation for Mt. Vesuvius, Campi Flegrei, and Ischia. (**A**) Likelihood for the threshold interevent time τ (black dotted line), the most likely estimation (MLE) value and its confidence intervals (gray areas), and observed repose time (red dashed line). (**B**) Mean annual rates for low- and high-activity states (λ*_L_* and λ*_H_*, respectively): the left axis (in blue) reports the mean annual rates, while the right axis (in yellow) reports its equivalent average return period (evaluated as 1/λ). Error bars indicate the 70% confidence interval.

The quantification of the parameters for the three volcanoes is performed over the part of the eruptive histories that may be assumed reasonably comparable with its most recent activity, that is, the last 2000 years for Mt. Vesuvius (*46*), the last 14,000 years for Campi Flegrei (*41*, *49*), and the last 3000 years for Ischia (*51*, *53*). Parameters are estimated on the basis of the maximum likelihood estimation (MLE). More details on eruptive histories and parameter estimation are discussed in Materials and Methods. The results are reported in Table 1 and Fig. 3.

**Table 1.** MLE parameters. MLE parameters for Mt. Vesuvius, Campi Flegrei, and Ischia. To increase readability, we report both mean annual rates λ*_L_* and λ*_H_* and their reciprocals 1/λ*_L_* and 1/λ*_H_*, that is, the corresponding mean return periods.

**Parameter**	**Mt. Vesuvius**	**Campi Flegrei**	**Ischia**
λ*_L_* (year^−1^)	8.11 × 10^−3^	4.76 × 10^−4^	2.85 × 10^−3^
1/λ*_L_* (year)	123	2100	352
λ*_H_* (year^−1^)	4.81 × 10^−2^	1.00 × 10^−2^	1.39 × 10^−2^
1/λ*_H_* (year)	21	100	72
τ (year)	61	287	197

For Mt. Vesuvius, the threshold interevent time τ is estimated in the order of decades. In contrast, for both Campi Flegrei and Ischia, τ is estimated in the order of centuries. Comparing τ to the ongoing repose times, we can quantitatively check whether a volcano is presently in high or low activity. The likelihood distribution of τ (Fig. 3A) provides a quantitative estimation of how likely it is that the present repose time is actually larger than τ, estimating our confidence in the present state of the volcano. Our results show that very likely all the three volcanoes are in a low-activity state: considering the uncertainty on τ, evaluated through the so-called profile-likelihood confidence region estimation (*58*) (see Materials and Methods), we see that the ongoing repose times are outside the 70% confidence interval (equivalent to 1 σ) for all the volcanoes and inside the 95% confidence interval (equivalent to 2 σ) only for Mt. Vesuvius.

The results show a good separation between the mean annual rates of high- and low-activity states, for all volcanoes, also considering the uncertainty [evaluated as suggested by Ross (*59*), see Materials and Methods]. The estimated annual rates for low- and high-activity states are separated by approximately one order of magnitude for all the three volcanoes (Fig. 2B). Notably, again, Mt. Vesuvius shows quicker dynamics, with annual rates larger than those of Campi Flegrei and Ischia in both low- and high-activity states, and even low-activity annual rates comparable to the high-activity annual rates at the other two volcanoes.

### Comparison with observations

**Fig. 4.**
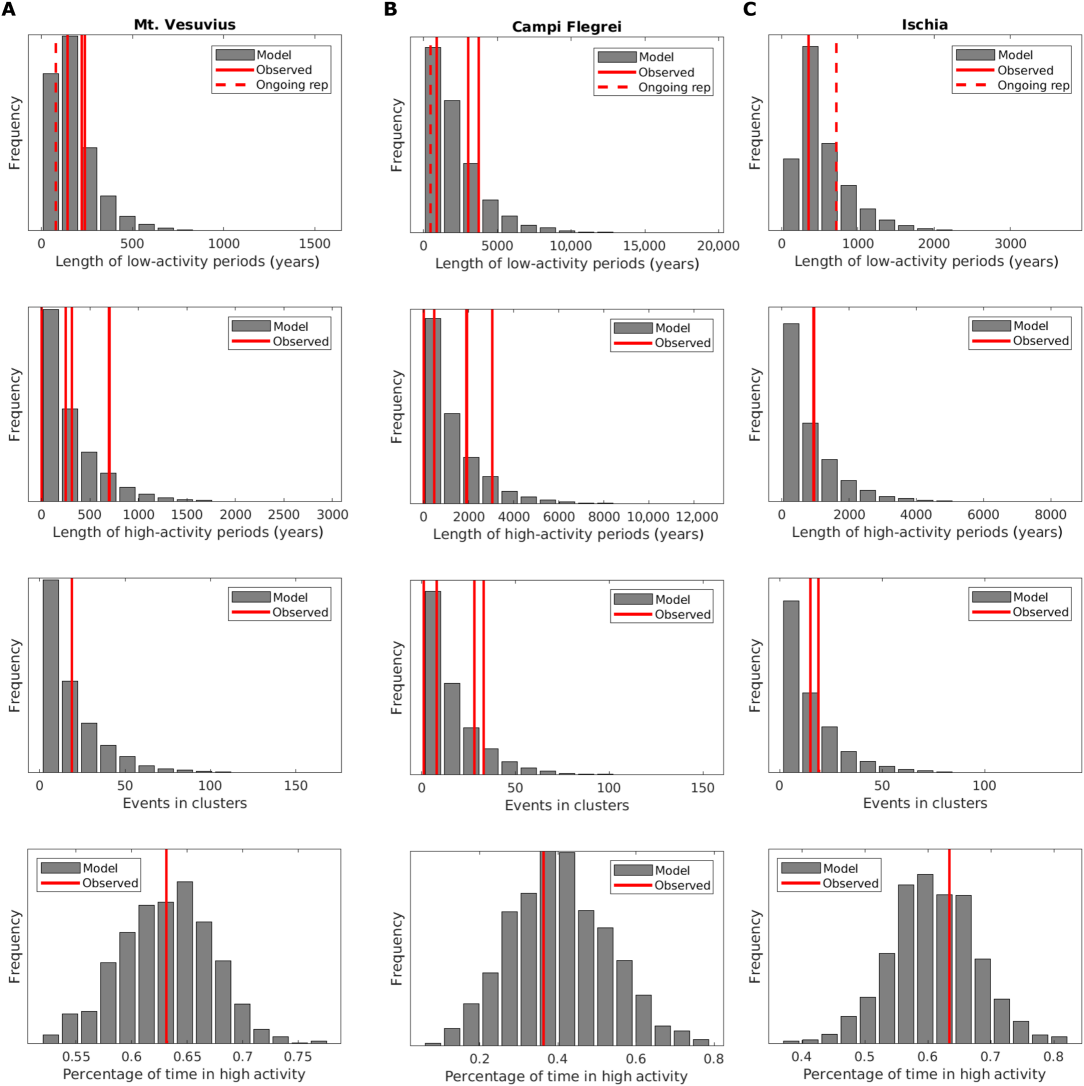
Comparison with observations. We compare observations (red lines) with the distributions obtained from 1000 synthetic catalogs generated by the model. We compare, from top to bottom, the duration of periods in low- and high-activity states, the number of eruptions in each cluster, and the percentage of times the volcano is in high activity for (**A**) Mt. Vesuvius, (**B**) Campi Flegrei, and (**C**) Ischia. The existence of multiple periods of low or high activity at each volcano generates multiple observations, corresponding to multiple solid red lines. With a red dashed line, we also report the present-day repose time (time from last eruption), which may be compared with the length of periods in low activity. In fig. S8, we report the same plots but using the empirical cumulative distribution function, to better compare observations with percentiles.

To evaluate the performance of the model, we compare the statistics of the real and synthetic catalogs generated by sampling the model with parameters fixed at the MLE values (see Materials and Methods). Being the parameters of the distributions used to produce the synthetic catalogs estimated by the same observations, the rationale of this comparison is to make a sanity check to verify that all the main characteristics of the real catalogs are well captured by this simple three-parameter model. As observables, we consider the duration of periods in low- and high-activity states, the number of eruptions in each cluster, and the percentage of time that the volcano is in high activity (Fig. 4). The comparison between observed values and the distributions obtained with the 1000 alternative synthetic catalogs generated by the fitted model is satisfactory, as there are no observations that fall in the tails of distributions. The percentage of time in high/low activity is close to the mode of distributions. For all the other statistics, there are multiple observations for each volcano, and thus, we can apply a statistical test (a one-sample Kolmogorov-Smirnov test) to quantify the distance between observations and the empirical distribution derived from the synthetic catalogs. The results (Table 2) show that the null hypothesis of equal distribution cannot be rejected, demonstrating that observations and synthetic catalogs are fully compatible. Notably, all the three volcanoes show a nonnegligible probability of having isolated events, i.e., eruptions opening a high-activity period that lead to no further events. In particular, the probability that a high-activity period ends with only one event (just the eruption that opened the period) is 0.06, 0.06, and 0.07 for Mt. Vesuvius, Campi Flegrei, and Ischia, respectively: These values are computed as the fraction, among the high-activity periods in the generated synthetic catalogs for each volcano, of the single-event high-activity periods. By dividing the number of isolated events by the length of the synthetic catalogs, we obtain the mean annual rate of isolated events, or equivalently its reciprocal, the mean return period. We obtain a mean return period of isolated events of 8900 years for Mt. Vesuvius, 68,700 years for Campi Flegrei, and 20,300 years for Ischia. These values are compatible with the observations, that is, one event in 2000 years for Mt. Vesuvius (the AS5 medieval event), one in 14,000 years for Campi Flegrei (the Monte Nuovo eruption), and none in 3000 years for Ischia: By assuming Poisson distributions with these mean return periods, the probability of having at least one isolated eruption in 2000 years at Mt. Vesuvius and in 14,000 years at Campi Flegrei is about 0.20 and 0.18, respectively, while the probability of having no isolated events in 3000 years at Ischia is 0.86. Therefore, also the observation of such isolated events at Mt. Vesuvius and Campi Flegrei and none at Ischia is compatible with the model.

**Table 2.** Test results. *P* values of the Kolmogorov-Sminov one-sample test. We select the significance level of 0.05 for the test; values larger than 0.05 indicate that the null hypothesis of equal distributions cannot be rejected, indicating a good performance of the model.

	**Mt. Vesuvius**	**Campi Flegrei**	**Ischia**
Low-activity length	0.34	0.52	0.74
High-activity length	0.72	0.86	0.19
Number of events in high-activity periods	0.70	0.68	0.24

**Fig. 5.**
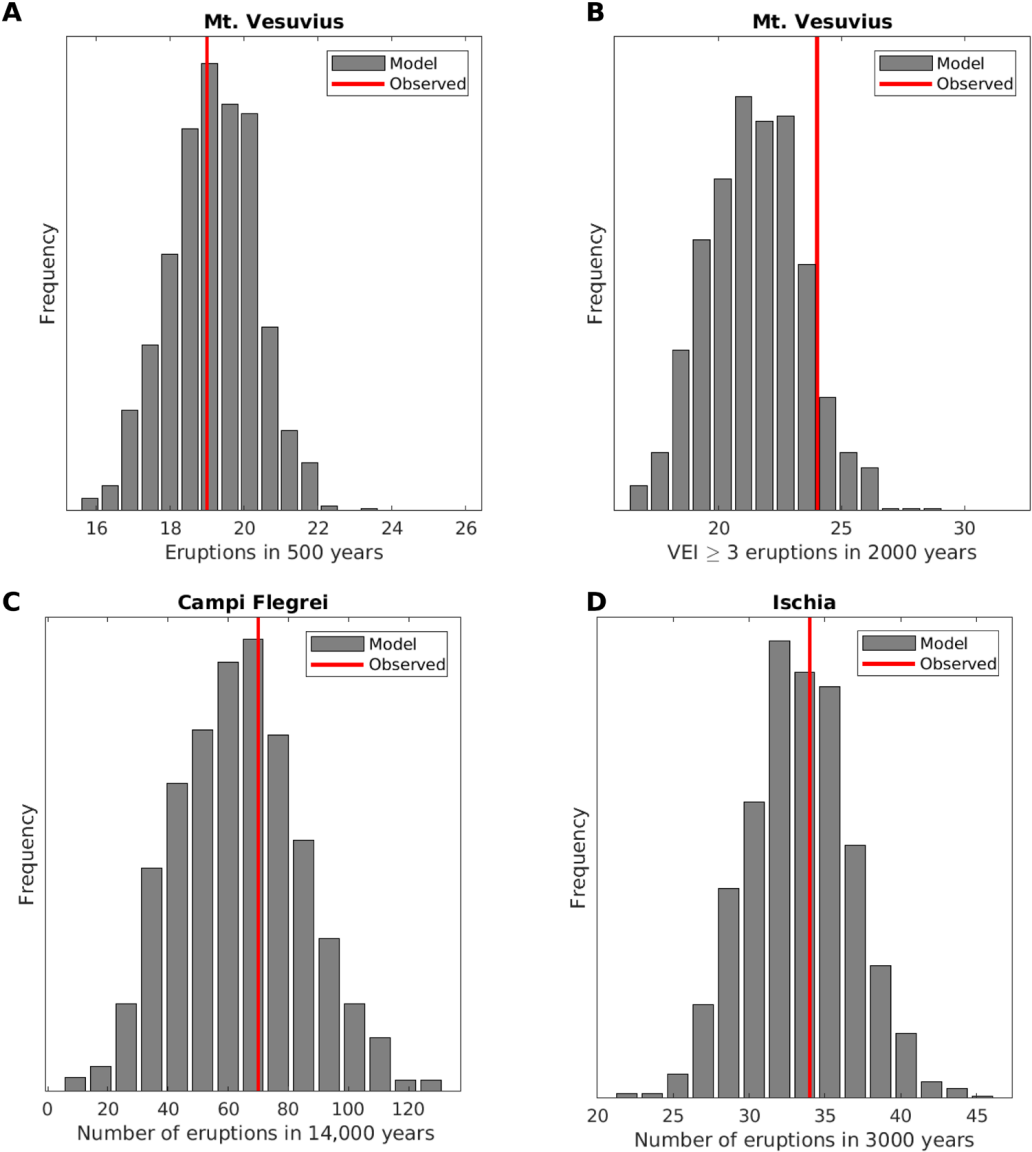
Comparison with number of eruptions. (**A**) Comparison between observations (red lines) and the distributions obtained from 1000 synthetic catalogs generated by the model (gray columns) for Mt. Vesuvius, considering all eruptions in the last 500 years. (**B**) Same as (A) but considering only VEI ≥ 3 eruptions, for which the catalog may be extended up to 2000 years. (**C**) Same as (A), for Campi Flegrei, considering all eruptions in the last 14,000 years. (**D**) Same as (A), for Ischia, considering all the eruptions in the last 3000 years.

In Fig. 5, we report the comparison of simulated and observed number of eruptions. To this end, we scale the statistics with the length of the complete part of the real catalogs, that is, for Mt. Vesuvius, 500 years for all eruptions and the entire period of 2000 years for eruptions with volcanic explosivity index (VEI) ≥ 3 only, while for Campi Flegrei and Ischia, the entire periods of 14,000 and 3000 years, respectively (see Materials and Methods for more details). As said, for Mt. Vesuvius, the check is performed also considering a subset of eruptions (the ones with VEI ≥3) in the entire period of 2000 years. To simulate VEI ≥ 3 synthetic catalogs, we must also set the frequency-magnitude distribution for Mt. Vesuvius. Following Marzocchi *et al.* (*37*), we define two different distributions for low- and high-activity states (see Materials and Methods). In this way, we can sample synthetic catalogs randomly assigning a specific size to each eruption according to the specific frequency-magnitude distribution, and then we can compute the distribution of the number of eruptions of any predefined size interval. For all volcanoes, the real observations are very close to the modes of distributions, showing again full compatibility of synthetic catalogs and data.

Last, we check the compatibility between the observed interevent times δ*t* and τ. On the basis of our conceptual model, we expect dt < τ within high-activity states, and dt > τ between them. This is indeed the case, with a few exceptions. For Campi Flegrei (where τ = 287 years, see Table 1), within epochs dt are smaller than τ, with the exception of the last eruption of epoch 3 (Nisida). However, the time for this eruption is rather uncertain in the catalog, also because it is unconstrained by the stratigraphy, and a shorter interevent time is clearly possible (Fig. 1A). For Ischia (where τ = 197 years, see Table 1), the dt preceding the Cretaio eruption [250 years in (*53*)] is larger than τ. However, also in this case, the uncertainty on this dt is very large: most of the eruption dates are rounded to 50 years (*53*), including the Cretaio eruption (ca. 1950 BP) and the two eruptions preceding it (both ca. 2200 BP). In any case, the stability of the results has been checked both for Campi Flegrei separating Nisida from epoch 3 and for Ischia separating Cretaio from the first period of high activity (see Supplementary Text and figs. S1 to S4).

### Eruption probability

**Fig. 6.**
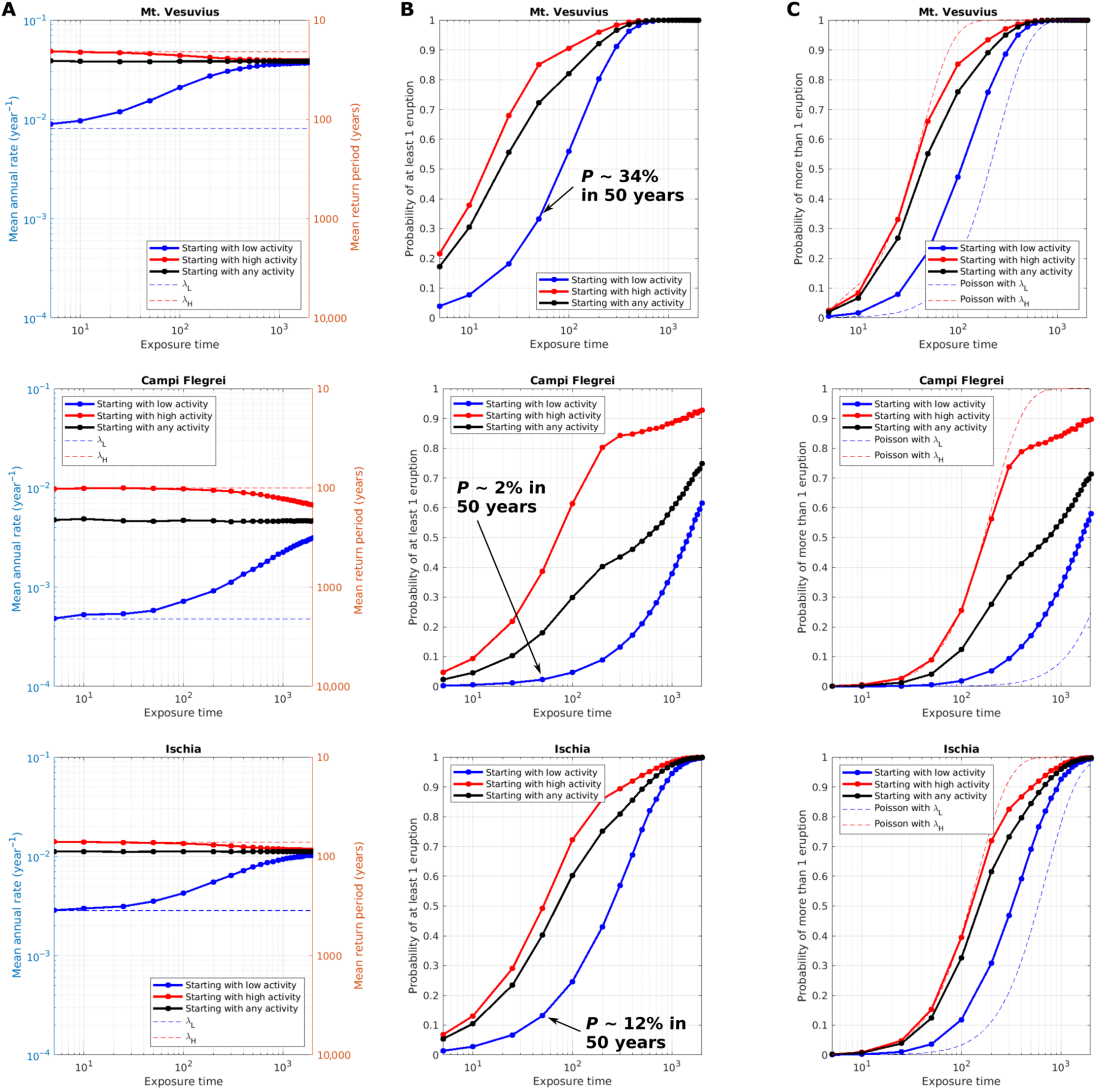
Average annual rates and probability of eruption. (**A**) Mean annual rates, (**B**) probability of at least one eruption, and (**C**) probability of more than one eruption, for different time intervals Δ*T*, independently from the state of the volcano at the beginning of the time interval (black lines), for time intervals starting with low activity (blue lines), and for time intervals starting with high activity (red lines). The probability of multiple eruptions (C) is compared with the standard stationary Poisson model with parameters λ*_L_* and λ*_H_*, which characterize the starting point for low- and high-activity states, respectively. To magnify differences for small probabilities, the same plots are reported in logarithmic scale in fig. S5.

The possibility of applying the same model to different volcanoes enables a meaningful comparison of their probabilities of eruption and consequent volcanic hazards. The developed model is a nonhomogeneous Poisson model that is characterized by a time-dependent mean annual rate λ(*t*). As for all Cox-Hawkes processes (*41*, *55*, *56*), characterized by self-exciting processes, the average number of events and the average mean annual rate $\bar{\lambda (\Delta T)}=\bar{n_{\Delta T}}/\Delta T$ can be evaluated using the synthetic catalogs, simply counting and averaging the number of events in time intervals of equal length (see Materials and Methods). The results are reported in Fig. 6A, for Δ*T* ranging from 5 to 2000 years.

When we take all time intervals independently from the initial state (either high or low activity; black lines in Fig. 6), $\bar{\lambda }$ is independent from Δ*T* and equals the weighted average of λ*_L_* and λ*_H_*, where weights are the fraction of time that each volcano remains in low- and high-activity states, respectively (reported in Fig. 4). If instead we assume to know the state of the volcano at the beginning of each Δ*T*, the average annual rates do change significantly. In Fig. 6A, we report the evaluation of $\bar{\lambda (\Delta T)}$ keeping separated the time intervals that start with low- or high-activity states (blue and red lines, respectively). The results show that, if Δ*T* is short enough, it is possible to assume that the volcano will not change its state during Δ*T*, so that $\bar{\lambda }$ tends to λ*_L_* or λ*_H_*, for periods starting with low or high activity, respectively. The longer the Δ*T*, the larger the probability that the volcano will change state, so that $\bar{\lambda }$ progressively tends toward its overall average. The transition between the two regimes depends on the volcano, being quicker for Mt. Vesuvius and slower for Ischia and Campi Flegrei (Fig. 6A).

Similarly, the probability of eruption also depends on Δ*T* and in the initial state (high or low activity). In Fig. 6 (B and C), we report the probability of observing at least one or more than one eruption, respectively, again for Δ*T* ranging from 5 to 2000 years, either considering the initial state in each time interval separately or not. As expected, the probability of eruption strongly depends on the initial state. The probability values applicable for each volcano at any time *t* may be defined by judging the state of the volcano at time *t*, that is, by comparing the ongoing repose time with the maximum likelihood τ, as discussed above. Alternatively, an averaged curve falling between these extremes may be defined by accounting for the epistemic uncertainty on the current state of the volcano (see Materials and Methods). In any case, the difference in probability between time intervals starting with low-/high-activity states tends to decrease with increasing Δ*T*. Notably, over the shortest Δ*T* (5 years), only Mt. Vesuvius has a nonnegligible probability of eruption, while over the longest time interval (2000 years), the probability of observing at least one eruption is almost 1 for both Mt. Vesuvius and Ischia, and it is significantly <1 for Campi Flegrei only.

The possibility to switch between the states, which is recorded by the increase/decrease of the average mean annual rates of Fig. 6A, has a significant impact in the probability of observing more than one event in Δ*T*, also for relatively small exposure time intervals. In Fig. 6C, we compare this probability with the one that is obtained by assuming a stationary Poisson model with λ*_L_* or λ*_H_*, which corresponds to the initial conditions for periods that start in low- and high-activity state, respectively: Already for Δ*T* of a few decades, a standard Poisson model with λ*_L_* significantly underestimates the probability of multiple eruptions in a period starting in low activity. This is due to the fact that, as seen above, only about 5% of high-activity periods end with only one isolated eruption, which implies that 95% of such periods record multiple eruptions. Similarly, a stationary Poisson model with λ*_H_* overestimates this probability in periods starting in high activity already for Δ*T* of a few decades, because relative short low-activity periods are also possible in our model.

### Multivolcano volcanic hazard

The adopted occurrence model gives us the possibility to produce a homogeneous estimation of the long-term volcanic hazard from the Neapolitan volcanoes. In the literature, volcanic hazard is often computed either conditional to an eruption (i.e., without computing the probability of the eruption) or by multiplying the conditional hazard by the probability of an eruption. The latter assumes that more than one event in the exposure time is unlikely, but Fig. 6C shows that, for typical exposure times in long-term hazard quantification [tens of years to centuries or more (*31*)], this is not always the case. For example, the probability of more than one eruption in 50 years falls within the range of 0.22 to 0.67 for Mt. Vesuvius, that is, 0.22 if Mt. Vesuvius is assumed in a low-activity state and 0.67 if it is assumed in a high-activity state. If the epistemic uncertainty on τ is considered, the probability estimates fall between these two extremes. Similarly, such probability ranges between 0.005 and 0.10 for Campi Flegrei and between 0.03 and 0.16 for Ischia, for time windows starting with low or high activity, respectively. Thus, for volcanic hazard assessment in Naples, it is fundamental to account for possible multiple eruptions in the exposure time and not only for the next eruption, given that the probability of multiple eruptions is not negligible. To do this, the hazard can be evaluated by multiplying the hazard conditional upon a specific eruption size and its mean annual rate in the exposure time (see Materials and Methods).

**Fig. 7.**
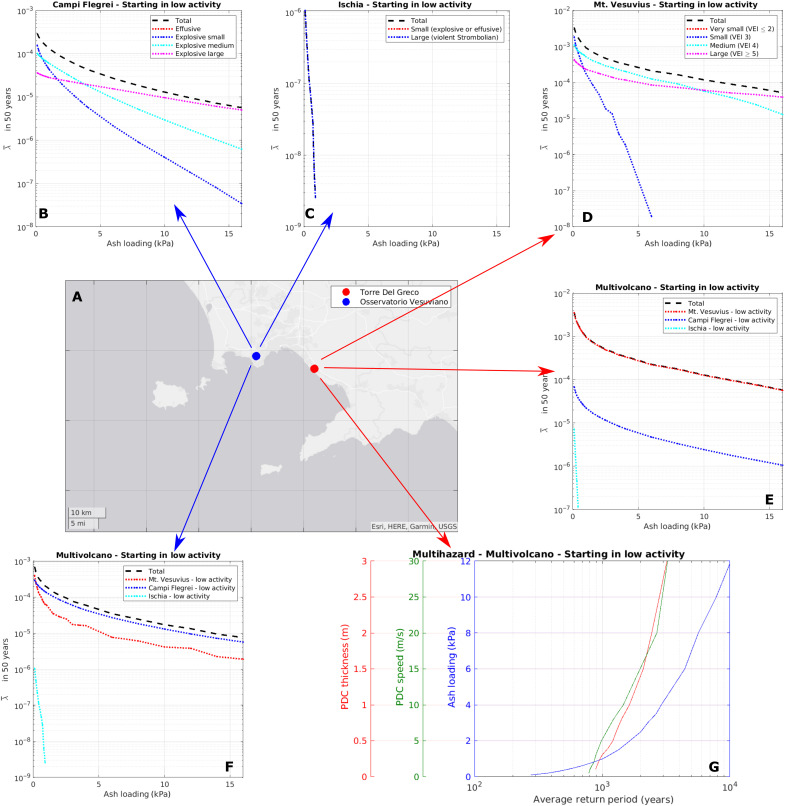
Hazard curves. (**A**) Target points. (**B**) Hazard curves for tephra fall from Campi Flegrei, computed at OV for an exposure time Δ*T* of 50 years and assuming an initial low-activity period, and the contribution of each size. (**C**) Same as (A), for Ischia. (**D**) Same as (A), for Mt. Vesuvius and at TdG. (**E**) Total hazard curves in TdG (Δ*T* = 50 years) from all the volcanoes and respective contributions. (**F**) Same as (E), but in OV. (**G**) Hazard curves in TdG (Δ*T* = 50 years) for tephra fall, dense PDC speed, and thickness, reporting the hazard intensity as a function of the average return period.

In Fig. 7, we report the hazard curves for tephra fallout in two positions within the Bay of Naples for Δ*T* = 50 years. To this end, the mean annual rates of the different sizes are multiplied by the tephra fallout hazard curves conditional to an eruption of a given size (see Materials and Methods). Such conditional hazard curves are taken from Sandri *et al.* (*34*) for Campi Flegrei and Mt. Vesuvius and from Stocchi *et al.* (*60*) for Ischia. In Fig. 7, we compare and sum the contribution of the different sizes to the total absolute hazard curve for tephra fallout for each individual volcano. For Mt. Vesuvius, the hazard curve is evaluated in the area of Torre del Greco (TdG), at the bottom of the western flank of Mt. Vesuvius (Fig. 7D). In this area, the hazard curve is dominated by eruptions of medium size (VEI 4) in the range between 1 and 10 kPa, being the contribution of this size to the total hazard the largest among the considered sizes in this range (Fig. 7D). With loading >10 kPa, it is instead dominated by larger (VEI 5+) eruptions, and smaller (<1 kPa) loadings are dominated by smaller (that is, VEI ≤3) eruptions. For Campi Flegrei and Ischia, hazard curves are calculated at the location of the Osservatorio Vesuviano (OV), within the Campi Flegrei caldera (Fig. 7, B and C, respectively). For Campi Flegrei, the results are similar to Mt. Vesuvius, but the interval in which the medium explosive events dominate is smaller and limited to approximately 2 to 4 kPa. For Ischia, there is only one size contributing (Cretaio-like), with a relatively small hazard in the target point (∼20 km northeast of Ischia).

The homogeneous formulation of the temporal model allows stacking and meaningfully comparing the hazard generated by more than one volcano, as well as by more than one hazardous phenomenon, in a multivolcano and multihazard perspective. In Fig. 7 (E and F), we evaluate the total hazard for tephra fall combining the three volcanoes at TdG and OV, respectively. As expected, at TdG, the dominant contribution is from Mt. Vesuvius, while at OV, the dominant contribution is from Campi Flegrei. This means that, even though the probability of eruption at Campi Flegrei is much smaller than at Mt. Vesuvius, the probability of tephra fallout westward from a Mt. Vesuvius eruption is even smaller due to the statistical distribution of winds (*33*), and the net effect is that Campi Flegrei still dominates the hazard. In comparison, the hazard posed by Ischia is instead negligible in these target points.

At TdG, we also report a multihazard comparison (Fig. 7G). Here, we report hazard curves exchanging the axes, and in the *x* axis, we report the average return period (which is $1/\bar{\lambda }$ for Δ*T* = 50 years), while we use multiple *y* axes to report the hazard intensity corresponding to different hazardous phenomena. As an example, in the figure we report the hazard from tephra fallout from all volcanoes (as above) and from dense pyroclastic density currents (PDCs) from Mt. Vesuvius, the latter in terms of maximum flow thickness and speed. Both PDC hazard curves are computed considering the hazard curves conditional to an eruption of a given size from the crater of Mt. Vesuvius evaluated by Tierz *et al.* (*36*). The graph in Fig. 7G represents a multihazard comparison, allowing us to easily compare the intensity of the different hazards corresponding to the same average return period. For example, at the 2000-year average return period, we find, approximately, a tephra fall loading of 2 kPa, a dense-PDC thickness of 1.25 m, and a dense-PDC speed of 15 m/s.

## DISCUSSION

We have shown that the proposed conceptual model, even in its simplest possible implementation with a Poisson model in both states, is able to satisfactorily reproduce all the main characteristics of existing eruption catalogs for the three volcanoes of Naples. Notably, the model not only reproduces the sequence of eruptive clusters but also allows accounting for isolated eruptions and long-term repose times, which are possible for all the three volcanoes. The application of the model requires the knowledge of the eruptive history, including at least the complete record of one cycle and a definition of existing clusters. This is possible for well-studied volcanoes such as Mt. Vesuvius, Campi Flegrei, and Ischia and may be achievable for many other volcanoes worldwide. At other, data-scarce Holocene volcanoes [e.g., (*61*)], the application of our model may be more challenging, yet achievable by promoting the acquisition of new data, as well as by making use of data or experience obtained from analogue volcanoes [e.g., (*62*)]. Further applications and testing of our model will help better understand its generality and discuss potential required sophistications.

Being connected to a specific conceptual model, the obtained parameters may help to improve our volcanological interpretation of the target volcanoes. The mean annual rate λ*_L_* quantifies the time requested for passing from a low- to a high-activity state, which is governed by the time needed for restoring the overpressure conditions for erupting magma. These conditions comprise the parcel of pressure needed for rupture of the host rocks and to sustain the local confining stress. The overpressure restoration may be attained through arrival of fresh magma into the feeding system or lowering the lithostatic stress acting on the magma batch. In the case of eruptions fed by the shallow feeding system (shallower than 5 km), the lowering of lithostatic stress is mainly limited to the occurrence of volcanic edifice failure (*23*). The annual rate λ*_H_* quantifies the readiness to erupt of a perturbed system: a system in high activity, which is typically weakened by recent eruptions and potentially characterized by availability of magma at shallow levels. Thus, λ*_H_* measures the level of fragility of the system during high activity, which may be controlled by the existence of open conduits or, more in general, by the general conditions in stress that may promote the pathway toward the surface for incoming magma batches. The interevent time τ is instead connected to the dominant process in restoring the equilibrium in the system, which is controlled by processes such as plugging of conduits, variation of stress conditions controlling magma pathways, cooling and/or crystallization of magma, and degassing.

Mt. Vesuvius shows quicker dynamics than the other volcanoes, with higher annual rates in both high- and low-activity states, as well as a more rapid exit from high-activity periods. This reflects the observations in the time window of activity used to train the model, characterized by prolonged periods of open-conduit dynamics, in which the magma is easily transferred toward the surface (*4*–*6*), sometimes accompanied by the emptying of the shallower feeding system (*63*). Edifice failures at Mt. Vesuvius are neither reported in historical chronicles nor deducible from geological record (*46*). This suggests that the opening and the closure of cycles during the last 2000 years was fully driven by balance between injection and eruption of magma from the shallow feeding system. We found that these conditions recur with an average return period of about 20 years in open-conduit conditions and of about 120 years in closed-conduit conditions. Assuming a constant feeding rate, the opening eruption of a cycle should be the largest in the cycle, as it is preceded by the longest repose times, as it is actually observed for both AD 472 and AD 1631 eruptions. In contrast, the open-conduit activity is characterized by lower magnitude-intensity distribution of eruptions. This implies the need for different frequency-magnitude distributions in low- and high-activity periods (*34*, *37*). Notably, some authors (*64*) also noted that the last eruption of each cycle is relatively larger than the others inside the cycle (excluding the opening eruption), which, within a time-predictable framework with constant feeding, would correspond to a longer interevent following the last eruption in a cycle (*4*, *11*), as foreseen by our model. The threshold interevent time τ is in the order of tens of years, demonstrating that the system also blocks relatively quickly. The most probable mechanism for this blockage, which characterizes the return to low-activity periods, is the closure of the feeding system due to magma cooling, which is characterized by similar characteristic times (*65*).

Campi Flegrei shows lower activity rates than Mt. Vesuvius and a much longer threshold interevent time τ, in the order of hundreds of years. The identification of such a slower dynamics is possible because the spatial spreading of the volcanic activity favors the identification of past eruptions in a much longer time window. Notably, the high-activity state is characterized by an annual rate similar to the one of Mt. Vesuvius during low-activity periods (about 100 years in terms of average return period), highlighting the relative concept of high-/low-activity states. Campi Flegrei’s longer characteristic times highlight that the processes for entering/exiting high-activity states significantly differ between the two volcanoes. The longer time scales of Campi Flegrei probably reflect the time needed for reestablishing overpressures in a volcanic system larger than Mt. Vesuvius, where the lack of large volcanic edifices implies the need to open new pathways toward the surface for each magma intrusion (*66*). This is consistent with the observation that in large calderas, many magmatic unrest episodes (possibly due to movement of magma at shallow layers) do not end in eruptions, with a high percentage of “failed eruptions” (*1*, *67*, *68*). This is also consistent with the fact that, differently from Mt. Vesuvius, it is not possible to define a different frequency-magnitude distribution for eruptions occurring at the beginning or during eruptive epochs (*49*). The driving process during high-activity states at Campi Flegrei is not the formation of open-conduit conditions, but it is controlled by the availability of a sufficiently large amount of eruptible magma at shallow levels, particularly below areas where eruptions may be triggered by relatively small changes in the local stress. They could be also favored by the failure of the roof of a shallow magma reservoir, which, in turn, may also be promoted by the release of H_2_O-rich and hot magmatic gasses, which can heat the hydrothermal system and the shallower rocks and accelerate the deformation, ultimately culminating into an eruption (*69*). The occurrence of one eruption may favor such conditions, due to the additional structural loading of the newly formed volcanic edifices (*66*), generating new high-activity periods characterized by a disequilibrium condition very different from the one of Mt. Vesuvius. The new equilibrium at Campi Flegrei can be found only when the balance between the local magma pressure variations and the structural robustness of the feeding system is reestablished. The most likely mechanisms for this process are magma injection at depth, magma cooling, and crystallization, or changes in the stress conditions due, for example, to the tectonics, or the dynamics of the hydrothermal system, which affects the shallower rock layers.

Ischia shows long dynamics similar to Campi Flegrei, as expected from the polygenic nature of its volcanism (*51*–*53*). Ischian volcanism is dominated by caldera resurgence, with a relatively low impedance for rising magma to reach the surface, at least for magma bodies emplaced around the central most-resurgent block (*20*, *70*): Magma intrusions occurring below this central block fueled the resurgence without leading to eruptions, as testified by the total absence of eruptive events in the entire central block (*53*, *70*). This higher chance of failed eruptions for new magma batches rising from depth may be reflected in relatively low rates. Notably, Ischia has a rate of activity significantly larger than that of Campi Flegrei during low-activity states, showing that it has a relatively higher capability of entering new high-activity states, as testified by the larger percentage of time that Ischia passed in high activity (about 60 and 40%, for Ischia and Campi Flegrei, respectively; Fig. 4). This is probably connected to the relatively small size of Ischia eruptions (mostly <0.1 km^3^) and the fact that magma bodies far from the most resurgent block may feed multiple spatially clustered eruptions.

Last, from Figs. 2 and 4, we can see that the time spent in the high-activity state for three volcanic systems shows a significant variability, ranging from a few hundreds up to 1000 years. In particular, for Mt. Vesuvius, the length of such periods varies from 200 up to 500 years; for Campi Flegrei, it ranges from about 500 years up to 1000 years; and for the two cycles of Ischia, it is about 1000 years. Although the order of magnitude of the periods is similar, the different durations of the three volcanoes indicate that at calderas, once that equilibrium is broken, it is more difficult to reestablish it. However, it reflects the uniqueness of each volcanic setting, which underpins different feeding systems, local stress regime, and heat transfer to the surrounding environment. For example, the development of a significant hydrothermal system (more relevant for Campi Flegrei and Ischia with respect Vesuvius) can contribute to the deterioration and weakening of the shallow rock layers (*69*), contributing to a longer duration before reaching a new equilibrium state.

The proposed model allows estimating how likely it is that each volcano currently is in high or low activity, by comparing the ongoing repose times with the parameter τ. The ongoing repose times fall outside the 0.70 confidence interval (equivalent to 1σ) of the threshold interevent times for all the volcanoes, being outside the 0.95 confidence interval for both Ischia and Campi Flegrei. This indicates that it is almost certain that they all are in low-activity states, especially Campi Flegrei and Ischia. This has strong implications for evaluating present-day probability of eruption and consequent hazard, as we have shown that they strongly depend on the present state of the volcano.

The largest probabilities of eruption are estimated at Mt. Vesuvius, with a probability of eruption of ∼34% in 50 years for time intervals starting from low activity (as today). This high probability is compatible with what was found by other authors (*29*, *71*) and reflects the fact that Mt. Vesuvius has been in an open-conduit regime for more than 60% of the last 2000 years. Also, Ischia shows rather high probabilities, with a probability of eruption of ∼12% in 50 years for time intervals starting from low activity (as today). Notably, the current long repose time is totally compatible with our model, as it is included into the distribution of possible length of repose times for Ischia (Fig. 2), lying at the ∼75th percentile of the distribution. Thus, the model (and consequent probability of eruption) accounts for possible long quiescence periods, even longer than the present one.

The probability of eruption for Campi Flegrei in the present low-activity state is the smallest for the three Neapolitan volcanoes, with a probability of eruption of ∼3% in 50 years. This reflects the fact that the Campi Flegrei caldera is currently in a low-activity state and that, since eruptive epochs (i.e., high-activity periods) opened only four times in 14,000 years, such a switch has a rather small probability of occurring. As a consequence, the Campi Flegrei caldera shows the smallest percentage of time in a high-activity state (less than 40%). These probability values are significantly smaller than what was estimated by Bevilacqua *et al.* (*41*), who estimated probabilities of eruption between 10 and 50% in 50 years, under different hypotheses. This reflects the fact that their approach essentially concentrates in modeling interevent times inside epochs, accounting for both base return time and self-excitement inside these high-activity periods. This is equivalent to assume that Campi Flegrei are inside an epoch, and indeed the probability results of Bevilacqua *et al.* (*41*) are closer to the probability values that we find for periods starting with high activity.

Another important point to note is that our model does not consider the information contained in the monitoring data in the current state [e.g., (*38*, *71*)]. At present, this is particularly relevant for Campi Flegrei, which is currently in unrest. This is due to the fact that, as we do not know how many unrest episodes occurred during past low-activity states, we cannot differentiate the estimation of probability with or without ongoing unrest (*72*, *73*). What we note here is that, as a whole, our model clearly indicates that Campi Flegrei are in a low-activity period, in which the probability of eruption is rather small; as noted above, the existence of shallow magma bodies, which has been suggested by several authors as the cause of the present unrest (*74*), is not a sufficient condition for the onset of eruptions opening a high-activity period. If we could count the number of past unrest episodes during low-activity periods, it would be possible to differentiate between low-activity-with-unrest and low-activity-without-unrest substates, with higher probability of eruptions when the former state occurs. In this sense, our probability estimates average among these two hypothetical substates, thus representing a minimum probability to be eventually updated considering monitoring data and expert judgment (*38*, *71*, *75*).

Notably, the presented model is the simplest possible implementation of the proposed conceptual model. Such simplification derives from physical considerations applied to the mathematical model (quoting Bruno Munari, “To complicate is easy. To simplify is difficult.”). This feature is very appealing, as it is sufficient to interpret the eruptive history of three very different and well-studied volcanoes. Of course, specific adjustments and sophistications may be required to universally apply this model to other volcanic systems, such as the consideration of multiple states (*15*), or, for each state, the integration of temporal behaviors more complex than a homogeneous Poisson process (*12*). However, our application to the Neapolitan volcanoes highlights the need of homogeneous model definitions for an effective comparison among volcanoes and for producing coherent multivolcano long-term hazard and risk quantifications. The application of this concept, along with the inclusion of potential interaction between different hazardous phenomena (*45*, *76*), will represent an improtant step toward an effective inclusion of volcanic hazards in multihazard risk evaluations, toward the better understanding and the strengthening of disaster risk governance, as it is pursued through the Sendai Framework for Disaster Risk Reduction 2015–2030 of the United Nations Office for Disaster Risk Reduction (www.undrr.org/).

## MATERIALS AND METHODS

### Eruption time series and identification of high-/low-activity states for the Neapolitan volcanoes

High-activity states are tracked in the eruptive record by clusters of events separated from each other or by isolated events. Here, the identification of high-/low-activity states is mainly based on the scientific literature and compared with the results of a standard cluster analysis technique. The catalogs of eruptions adopted for Mt. Vesuvius, Campi Flegrei, and Ischia are reported in tables S1, S2, and S3, respectively.

The Mt. Vesuvius catalog (table S1) is derived from Cioni *et al.* (*46*). The age of the AS2 eruption has been selected to satisfy the stratigraphy, which places it after the AD 512 eruption. The catalog of eruption after AD 1631 can be assumed to be reasonably complete (*4*, *46*), considering the amount of historical chronicles. In the AD 79 to AD 1631 period, it can be considered reasonably complete only for major eruptions, that is, for VEI ≥ 3.

For Mt. Vesuvius, high-activity states are known mainly based on historical information, especially for recent (from the 17th century) and ancient (between 2nd century BC and 6th century AD) times. The periods adopted here derive from Cioni *et al.* (*46*), with three open-conduit periods after Pompeii, Pollena, and AD 1631 eruptions, and one isolated event approximately 600 years ago. These periods are coherent with the results of a hierarchical cluster analysis (HCA) with Euclidean distances. In fig. S6 (top), we report the dendrogram of this analysis, which shows that the first and most important division occurs between the 13th and the 14th eruptions (that is, between the end of the Pollena sequence and the isolated medieval event). The second most important division separates the medieval isolated event (AS5) from the AD 1631 activity, and the third division separates the Pompeii and the Pollena sequences.

The Campi Flegrei catalog of eruptions (table S2) is obtained by averaging the age of each event from 1000 synthetic catalogs that sample the epistemic uncertainty on eruption age and order. These catalogs are generated with the same criteria used by Bevilacqua *et al.* (*41*), which allows sampling the epistemic uncertainty on the eruption dates, and simultaneously to respect the stratigraphic order, where known. Here, we added a further constraint, that is, all eruptions in epoch 1 are forced to follow the Neapolitan Yellow Tuff caldera–forming eruptions, dated between 14,300 and 13,900 years ago (*77*): This adds a constraint that allows narrowing the date of the first two undated events in epoch 1, after sampling a random age for the Neapolitan Yellow Tuff within its age range. To check the stability of the results, we also considered an alternative catalog, in which the age of each event is again obtained by averaging its age from 1000 synthetic catalogs (see Supplementary Text and figs. S1 to S4). In this case, however, the synthetic catalogs are generated simply by sampling from the age ranges provided by Bevilacqua *et al.* (*41*) and respecting the stratigraphic order and the constraint deriving from forcing the eruptions in epoch 1 to be younger than the Neapolitan Yellow Tuff, whose age was again randomly sampled from its age range (*77*). In terms of number of eruptions, this catalog is assumed complete over the entire period (*41*, *48*, *49*).

For Campi Flegrei, high-activity periods have been set on the basis of the epochs of activity identified after the Neapolitan Yellow Tuff caldera formation (*41*, *49*) and consist of three epochs and one isolated event in AD 1538 (the Monte Nuovo eruption). The HCA’s dendrogram for Campi Flegrei is reported in fig. S6 (middle). The result shows that the first and most important divisions separate epoch 3 and Monte Nuovo Eruption from epochs 1 and 2, then Monte Nuovo from epoch 3, and finally the separation between epoch 1 and epoch 2. Other separations appear less relevant.

The Ischia catalog (table S3) is directly derived from Selva *et al.* (*53*), restricting to the last 3000 years, a period over which Ischia has shown a stationary behavior and the catalog can be assumed complete (*51*, *53*). For Ischia, a clear definition of high-activity periods is not available in literature, and thus, it is here entirely based on HCA results, reported in fig. S6 (bottom). The results show that the main division occurs between the Cretaio and the following eruption, separating two clusters: eruptions 1 to 19 and eruptions 20 to 34, respectively. This defines only two high-activity periods ending with the most relevant events in Ischia, the Cretaio and the Arso eruptions, which represent the largest explosive and effusive eruptions in the considered period (*51*–*53*). Further divisions are possible, but not clear, probably due to the very large uncertainty on the age of eruptions, which is rounded at multiples of 50 years (*53*). The largest apparent interevent time is observed before Cretaio (separating eruption 19 from eruptions 1 to 18).

### Parameter estimation

The parameters are evaluated through the MLE method. As input data, we consider the time of the eruptions, the list of clusters, and the membership of each eruption (i.e., to which cluster it belongs): {*t_i_*, *c_i_*}. The quantification is made assuming the independence of the estimation of the annual rates λ*_L_* and λ*_H_* and the one relative to τ. In practice, for a given τ, the periods during which the volcano is in a high-/low-activity state are fixed, the mean annual rate of high-activity periods λ*_H_* may be estimated as

$${\hat{\lambda }}_{H}=\frac{\text{no}.\;\text{of}\;\text{events}}{\text{duration of the period}}=\frac{\sum_{i}\;(n_{i}-1)}{\sum_{i}(\delta t_{i}+\tau )}$$ (1)

where *n_i_* is the total number of eruptions in cluster *i*, and δ*t_i_* is the duration of the cluster *i*, evaluated as the difference in time between the last and first eruption in the cluster. Each high-activity period starts with the first eruption in the cluster and ends τ after the last eruption, and ${\hat{\lambda }}_{H}$ is obtained by definition as the total number of observed eruptions (excluding the first, which ends the low-activity period) divided by the total time spent in a high-activity state. The uncertainty is estimated using the minimal-length integration of the Bayesian posterior (IBP) method [method 2 in (*59*, *78*)], which provides accurate estimates also for small datasets.

In analogy, the mean annual rate of low-activity periods λ*_L_* may be estimated as

$${\hat{\lambda }}_{L}=\frac{\text{no}.\;\text{of events}}{\text{duration of the period}}=\frac{n_{\text{CL}}}{T-\sum_{i}(\delta t_{i}+\tau )}$$ (2)

where *n*_CL_ is the number of clusters (only the first eruption in each cluster occurs in the low-activity period) and *T* is the total observational time in which the eruptive record may be considered complete, and it includes both low- and high-activity periods. In practice, low-activity periods are complementary to high-activity periods, and ${\hat{\lambda }}_{L}$ is obtained by definition as the total number of opening eruptions divided by the total time spent in low-activity periods. Also, in this case, the uncertainty is estimated using the minimal-length IBP method (*59*, *78*).

The parameter τ is estimated maximizing the log-likelihood of the observations {δ*t_i_*}, which is the value $\hat{\tau }$ that maximizes

$$\;\mathrm{LL}(\tau |\{\delta t_{i}\;\})=\sum_{j}\text{log}(g({\hat{\lambda }}_{H},\tau |\delta t_{j}))$$ (3)

where the likelihood $g({\hat{\lambda }}_{H},\tau |\delta t_{j})$is evaluated using a Monte Carlo approach. In practice, for a given τ, we generate *N* clusters by sampling the events exponentially distributed with parameter ${\hat{\lambda }}_{H}$ and evaluating the length of the cluster by comparing interevent times and τ. Then, we discretize potential cluster lengths in intervals of 10 years, and we evaluate $g({\hat{\lambda }}_{H},\tau |\delta t_{j})$ for each δ*t_j_* as the fraction of the sample in the interval corresponding to the observations δ*t_j_* with respect to the total numerosity of the sample *N*. For example, if δ*t_j_* = 321 years, and we sampled *n* clusters with length between 320 and 330 years of *N* samples for a specific couple of parameters ${\hat{\lambda }}_{H}$ and τ, the likelihood $g({\hat{\lambda }}_{H},\tau |\delta t_{j}=321)$ is equal to *n*/*N*. The convergence has been obtained with sample sizes of *N* of 20,000, 750,000, and 150,000, for Mt. Vesuvius, Campi Flegrei, and Ischia, respectively.

Uncertainty on the estimated $\hat{\tau }$ is evaluated using the so-called profile-likelihood confidence region estimation, a method already used in geophysical problems, which defines confidence intervals based on cuts in the likelihood distribution. This cutting threshold depends on the target confidence interval and can be computed on the basis of the chi-squared statistics (*58*). For example, the 0.95 confidence interval for log-likelihood distribution for one parameter can be estimated using the threshold for which the log-likelihood is equal to *LL*_max_ minus the 0.95 quantile of a chi-squared distribution with one degree of freedom (*58*). For simplicity, the estimated parameter values ${\hat{\lambda }}_{H}$, ${\hat{\lambda }}_{L}$, and $\hat{\tau }$ in the rest of the paper are simply indicated as λ*_H_*, λ*_L_*, and τ, respectively.

For Campi Flegrei and Ischia, the entire catalogs have been used to estimate the parameters. For Mt. Vesuvius, λ*_H_* is estimated considering only the complete part of the catalog (after the AD 1631 eruption), while λ*_L_* and τ have been estimated considering the count and length of high-activity periods in the entire period.

### Magnitude-frequency distributions

To refer as much as possible to literature estimations, magnitude-frequency distributions are here based on the Bayesian approach adopted in literature for the setup of the Bayesian Event Tree (BET) model [node 5; see (*71*)].

For Mt. Vesuvius, two different distributions are assumed for low- and high-activity states. The distribution for the low-activity states is the one characterizing the eruptions opening the clusters. In literature, it is assumed that these events should be from moderate to highly explosive eruptions (*37*). Specifically, three size classes are assumed, corresponding to VEI 3, VEI 4, and VEI5+. The posterior distribution is set by assuming a Dirichlet prior distribution with parameters [2.49, 0.42, 0.09], a multinomial likelihood with counting [4,2,1]. Consequently, the posterior distribution is a Dirichlet distribution with parameters [6.49, 2.42, 1.09].

The distribution for the high-activity states is not available in literature. To produce it, we also consider eruptions smaller than those considered by Marzocchi *et al.* (*37*). To this end, we add a new size class for eruptions smaller than VEI 3, and we set a Dirichlet prior distribution with parameters [1, 1, 1, 0], which is a noninformative prior distribution (uniform distribution) in which large Plinian eruptions (VEI5+) are considered practically impossible. The multinomial likelihood is based on counting in the complete part of the catalog, that is, after the 1631 eruptions, resulting in [15, 3, 0, 0] (table S1). Consequently, the posterior distribution is a Dirichlet distribution with parameters [16, 4, 1, 0]. The resulting distributions are plotted in fig. S7.

For Campi Flegrei, an equal frequency-magnitude distribution is assumed for both low- and high-activity states, as derived from literature (*34*, *35*, *49*). In particular, four eruptive size classes have been defined (effusive, explosive small, explosive medium, and explosive large). The posterior distribution is set by assuming a Dirichlet prior distribution with parameters [0.2, 3.16, 0.52, 0.12], a multinomial likelihood with counting [3, 15, 6, 2]. Consequently, the posterior distribution is a Dirichlet distribution with parameters [3.2, 18.16, 6.52, 2.12]. The resulting distribution is plotted in fig. S7.

Also for Ischia, an equal frequency-magnitude distribution is assumed for both low- and high-activity states. No estimations are available from literature. Being interested only in eruptions potentially generating tephra fall in the metropolitan area of Naples, we adopt a very simplified distribution, assuming only two size classes (small and large) and considering that only one eruption in the last 3000 years belongs to the largest size class [the Cretaio eruption; (*79*)]. With this assumption, the posterior distribution is a noninformative Dirichlet prior distribution with parameters [1, 1] and a multinomial likelihood with counting [33, 1] (*53*). Consequently, the posterior distribution is a Dirichlet distribution with parameters [34, 1]. The resulting distribution is plotted in fig. S7.

### Synthetic catalogs, annual rates, and probability of eruption

To test the model and to quantify the annual rates and probability of eruptions, we generate *N_s_* = 1000 synthetic catalogs with a Monte Carlo approach. Specifically, each catalog has a length of 25,000 years and starts with a low-activity period. Then, one interevent time is generated from an exponential distribution with mean annual rate λ*_L_*, and an eruptive size is assigned from the frequency-magnitude distribution relative to low-activity periods, that is, ϕ*_L_*(size). For the subsequent high-activity period, a sequence of interevent times are generated from an exponential distribution with mean annual rate λ*_H_*, and this sequence is kept until the first interevent time is larger than or equal to τ. An eruptive size is assigned to each eruption of the sequence from the frequency-magnitude distribution relative to high-activity periods, that is, ϕ*_H_*(size). Then, the system falls back to low activity, and the process is restarted. This loop is repeated until the target length of the catalog (25,000 years) is reached.

Annual rates and probability of eruptions are evaluated for a set of time windows, ranging from 5 to 2000 years. For the time window Δ*T* (exposure time, see Fig. 6), we considered the last *N_w_* (up to a maximum of 100) nonoverlapping time windows with duration Δ*T*, recording for the *i*th time window in the *j*th synthetic catalog the state opening the period (low or high activity), the number of eruptions for the *k*th size *n_ijk_*(Δ*T*), and the total number of eruptions *n_ij_*(Δ*T*) = ∑*_k_n_ijk_*(Δ*T*). The average annual rate and the probability of exceeding *m* eruptions for each Δ*T* (expliciting the dependence on the random variable for eruptions E) are evaluated, respectively, as

$$\bar{\lambda (E,\Delta T)}=\bar{n_{\mathrm{ij}}}/\Delta T=(\;\sum_{i=1}^{N_{w}}\sum_{j=1}^{N_{s}}\bar{n_{\mathrm{ij}}}(\Delta T))/(N_{w}*N_{s}*\Delta T)$$ (4)

$$P(E\geq m,\Delta T)=\sum_{i=1}^{N_{w}}\sum_{j=1}^{N_{s}}I(\bar{n_{\mathrm{ij}}}(\Delta T)\geq m))/(N_{w}*N_{s}*\Delta T)$$ (5)

where *N_s_* = 1000, and I(x) is an indicator function that takes the value 1 if *x* > 0, and 0 otherwise.

The average annual rates for specific sizes $\bar{\lambda (E_{k},\Delta T)}$ can be obtained from Eq. 4 substituting *n_ij_*(Δ*T*) with *n_ijk_*(Δ*T*). Similarly, average annual rates for specific initial states and specific sizes, $\bar{\lambda_{L}(E_{k},\Delta T)}$ and $\bar{\lambda_{H}(E_{k},\Delta T)}$ for low- and high-activity initial states, respectively, can be quantified by restricting the sum in Eq. 4 to the *N*′*_w_* < *N_w_* time windows that start at a specific state, that is, with low-/high-activity periods. In this way, we can quantify the average annual rates conditional to an initial low/high activity at the beginning of the time window. Notably, whatever is the initial state of the volcano, transitions among states during Δ*T* are possible.

The effective applicability of $\bar{\lambda_{L}(E_{k},\Delta T)}$ and $\bar{\lambda_{H}(E_{k},\Delta T)}$ to one specific volcano depends on the state of the volcano at the time of the estimation, which oscillates between the two activity states of the volcano (Fig. 1B). This state is not directly measured, but it may be evaluated comparing the time from the last eruption and the interevent time threshold τ. To account for the epistemic uncertainty on τ (Fig. 2) in a Bayesian sense, i.e., including the epistemic uncertainty on the present state of the volcano in the probability computation, the averaged annual rate can be computed as

$$\bar{\lambda (E_{k},\Delta T)}=p(L,t)\;\bar{\lambda_{L}(E_{k},\Delta T)}+p(H,t)\;\bar{\lambda_{H}(E_{k},\Delta T)}$$ (6)

$$P(E\geq m,\Delta T)=p(L,t)P_{L}(E\geq m,\Delta T)+p(H,t)\;P_{H}(E\geq m,\Delta T)$$ (7)

where *p*(*L*, *t*) and *p*(*H*, *t*) represent the probability of being at low- and high-activity state at time *t*, respectively, and *p*(*L*, *t*) + *p*(*H*, *t*) = 1. The rates $\bar{\lambda_{L}(E_{k},\Delta T)}$ and $\bar{\lambda_{H}(E_{k},\Delta T)}$ and the probabilities *P_L_*(*E* ≥ *m*, Δ*T*) and *P_H_*(*E* ≥ *m*, Δ*T*) represent estimates based on the maximum likelihood τ, while the expression in Eqs. 6 and 7 are the epistemic averages, that is, estimates that account for the epistemic uncertainty on τ, in a Bayesian perspective. For the three volcanoes in the Neapolitan area, which are all judged to be in low activity, incorporating this epistemic uncertainty results in a slight increase in rates and probabilities, even if the effective values will not differ significantly from $\bar{\lambda_{L}(E_{k},\Delta T)}$ and *P_L_*(*E* ≥ *m*, Δ*T*), as *p*(*H*, *t*) is, in all cases, quite low (i.e., Mt. Vesuvius results are outside the 0.70 confidence interval on the right tail, which corresponds to a probability of <0.15; Campi Flegrei and Ischia results are outside the 0.95 confidence interval on the right tail, which corresponds to probabilities of <0.025).

### Comparison with observations

We compared the statistics of synthetic and real catalogs considering as observable the number of eruptions, the length of periods in low- and high-activity states, the number of eruptions in each cluster, and the percentage of times that the volcano is in high activity. For simplicity, the lengths of high- and low-activity periods are compared without considering τ, that is, considering the duration of clusters {δ*t_i_*} evaluated as the distance in time between the first and last eruptions and the duration of repose times as the distance in time between the last eruption of the previous cluster and the first of the following one. In this way, these observations depend only on the catalog and not on the variable τ.

Observables from the real catalogs are evaluated considering the entire catalogs, as reported in tables S1 to S3. For Mt. Vesuvius, we tested two cases: all eruptions in the last 500 years, and VEI ≥ 3 eruptions in the last 2000 years. The number of VEI ≥ 3 eruptions is set by counting the eruptions in table 1 of Cioni *et al.* (*46*) for which a volume ≥ 0.01 km^3^ (corresponding to VEI ≥ 3) is reported, resulting in 24 eruptions (Fig. 5B).

### Quantification of hazard curves

Hazard curves report the mean annual rate of exceeding a specific value of the hazard intensity *X* (e.g., ash loading at ground) at a specific target site within the exposure time Δ*T*. For the time window Δ*T*, hazard curves for each single volcano are evaluated as

$$\bar{\lambda (x>X,\Delta T)}=\sum_{k}\bar{\lambda (E_{k},\Delta T)}\;P(x>X|E_{k})$$ (8)

where *P*(*x* > *X*∣*E_k_*) is the so-called conditional hazard curve reporting the probability of exceeding the intensity *X* at the target site if an eruption of size *k* occurs, and it depends on the propagation of the hazard from the source to the target; $\bar{\lambda (E_{k},\Delta T)}$ is the average annual rate of eruption with *k*th size, which depends on both annual rates of eruptions and frequency-magnitude distributions in both high- and low-activity states. As demonstrated in Fig. 4, $\bar{\lambda (E_{k},\Delta T)}$ may vary depending on the initial state of the volcano and the length of Δ*T*, due to the potential of changes in activity state during Δ*T*.

The hazard curves conditional to a specific initial state of activity, $\bar{\lambda_{L}(x>X,\Delta T)}$ and $\bar{\lambda_{H}(x>X,\Delta T)}$ for low and high activity, respectively, can be obtained by substituting in Eq. 8 $\bar{\lambda (E_{k},\Delta T)}$ with $\bar{\lambda_{L}(E_{k},\Delta T)}$ and $\bar{\lambda_{H}(E_{k},\Delta T)}$, that is

$$\bar{\lambda_{L}(x>X,\Delta T)}=\sum_{k}\bar{\lambda_{L}(E_{k},\Delta T)}\;P(x>X|E_{k})$$ (9)

$$\bar{\lambda_{H}(x>X,\Delta T)}=\sum_{k}\bar{\lambda_{H}(E_{k},\Delta T)}\;P(x>X|E_{k})$$ (10)

The conditional hazard curve depends only on the size of the eruption, and thus it is independent from the state of the volcano.

As in Eqs. 6 and 7, to account for the uncertainty on the state of the volcano, an averaged annual rate can be computed as

$$\bar{\lambda (x>X,\Delta T)}=p(L,t)\;\bar{\lambda_{L}(x>X,\Delta T)}+p(H,t)\;\bar{\lambda_{H}(x>X,\Delta T)}$$ (11)

Under the assumption of independence between the different volcanoes, the hazard curves, cumulating the contribution of the different volcanoes, can be obtained as the sum of the hazard curves of each volcano. At the time of the estimation, volcanoes may be at different states, and thus, the total hazard curve at a given time is obtained by summing the appropriate hazard curve for each volcano, which depends on the current state of the latter.

As discussed in section Multivolcano volcanic hazard, all the conditional hazard curves for Mt. Vesuvius, Campi Flegrei, and Ischia are taken from literature (*34*, *36*, *60*). In all cases, we neglect the epistemic uncertainty, and we report only the mean hazard curves. The reintroduction of epistemic uncertainty is straightforward, by substituting single estimations with ensembles of alternative estimates (*80*).
