# Supplementary material for: A simple two-state model interprets temporal modulations in eruptive activity and enhances multivolcano hazard quantification
Source: Sci Adv. 2022 Nov 2;8(44):eabq4415. doi: 10.1126/sciadv.abq4415 (PMC9629722; doi:10.1126/sciadv.abq4415)
Supplement: Supplementary file 1 — Supplementary Text Figs. S1 to S8 Tables S1 to S3 [file sciadv.abq4415_sm.pdf]

Science Advances
AAAS

Supplementary Materials for

A simple two-state model interprets temporal modulations in eruptive activity and enhances multivolcano hazard quantification

Jacopo Selva *et al.*

Corresponding author: Jacopo Selva, jacopo.selva@ingv.it

Sci. Adv. **8**, eabq4415 (2022)
DOI: 10.1126/sciadv.abq4415

This PDF file includes:

Supplementary Text
Figs. S1 to S8
Tables S1 to S3

Supplementary Text

The stability of results has been tested considering alternative definitions for both Campi Flegrei and Ischia. In particular, we recomputed all results by

1. separating, for Campi Flegrei, Nisida eruption from the rest of Epoch 3, as suggested by the comparison of inter-event times and the inter-event-time threshold τ (section Parameter Estimation);
2. merging, for Campi Flegrei, Epochs 1 and 2, as this is the less significant separation in the cluster analysis (section Eruption time-series and high/low activity periods);
3. considering, for Campi Flegrei, an alternative catalog in which only stratigraphic constraints are used to set eruption dates, as suggested by the analysis of the eruption sequence (section Eruption time-series and high/low activity periods).
4. assuming, for Ischia, that the Cretaio eruption is an isolated event, as suggested by the comparison of inter-event times and the inter-event-time threshold τ (section Parameter Estimation).

Notably, in these tests observations are moved from high to low-activity periods, or vice versa, adding/removing single events with impact on different parameters. This implies that our model is not sensible to small variations (few events) in the datasets. These changes are similar to what a few missing events due to catalog incompleteness would cause, allowing for indirectly testing the potential impact of incompleteness in the catalog.

All the results are reported in Fig. S1, S2, S3, and S4, for parameters estimation, catalog comparison, and probability quantification, respectively.

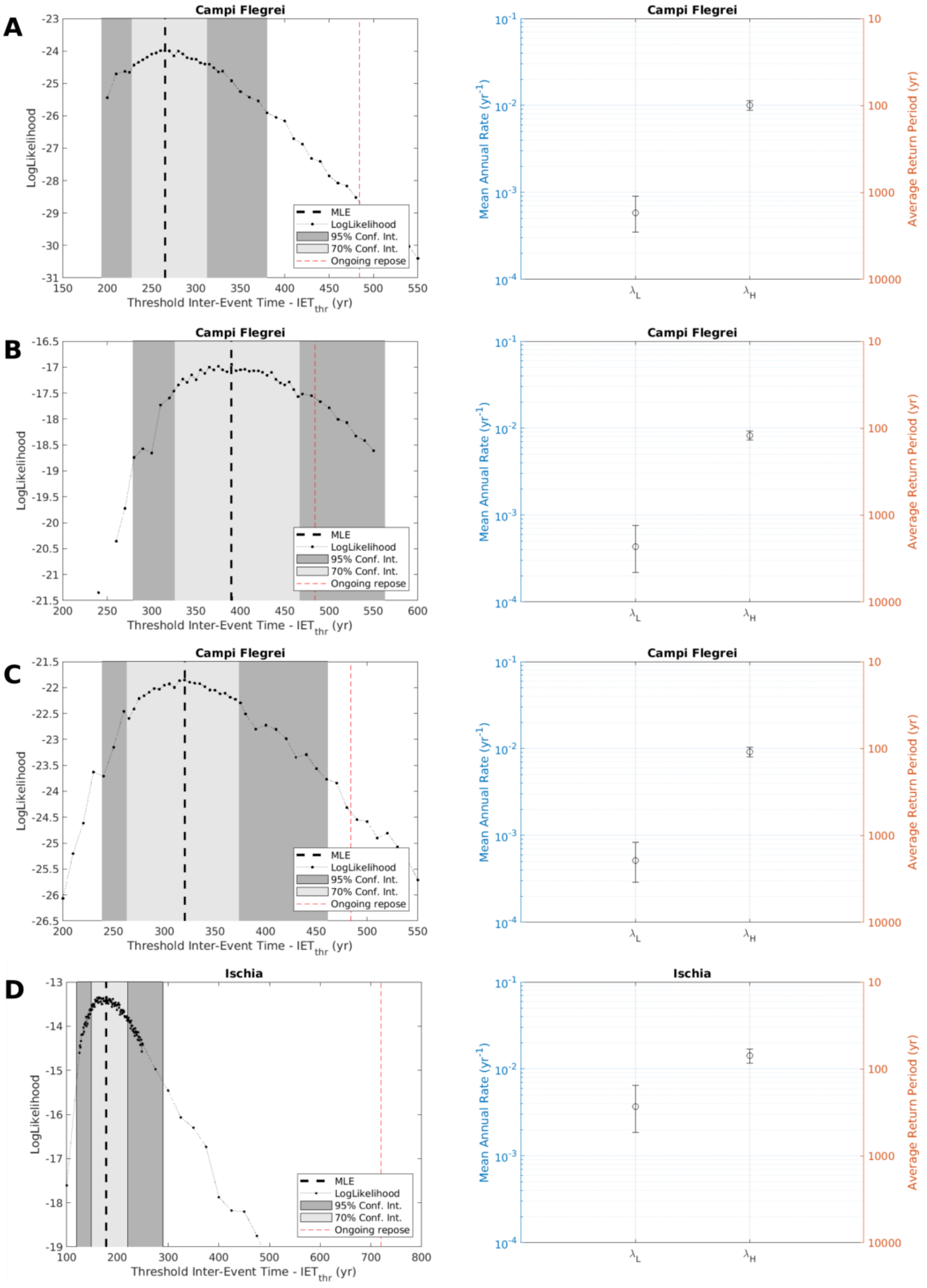

Fig. S1. Parameter estimation. Same of Fig. 2, but with modified clusters. For Campi Flegrei, we consider 2 alternative hypotheses. In (A), we consider the Nisida eruption separated from Epoch 3, ending up with five clusters, the last two being isolated events (Nisida and Monte Nuovo). In (B), we consider the catalog considering only stratigraphic constraints. In (C), we consider an alternative catalog for Campi Flegrei, in which only stratigraphic constraints are used to set eruption dates, as suggested by the analysis of the eruption sequence. In (D), for Ischia, we consider 3 clusters, separating the Cretaio eruption (cluster with only 1 event) from the rest of the previous eruption. All results are consistent.

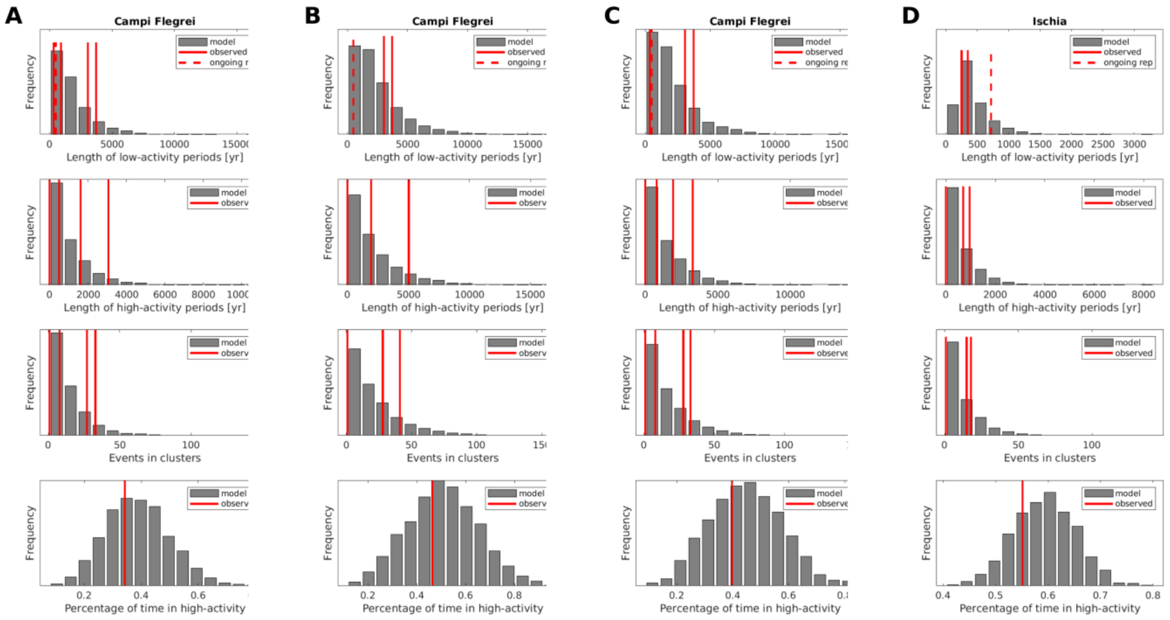

Fig. S2. Comparison with observations. Same of Fig. 4, but with modified clusters. For Campi Flegrei, we consider 2 alternative hypotheses. In (A), we consider the Nisida eruption separated from Epoch 3, ending up with five clusters, the last two being isolated events (Nisida and Monte Nuovo). In (B), we consider the catalog considering only stratigraphic constraints. In (C), we consider an alternative catalog for Campi Flegrei, in which only stratigraphic constraints are used to set eruption dates, as suggested by the analysis of the eruption sequence. In (D), for Ischia, we consider 3 clusters, separating the Cretaio eruption (cluster with only 1 event) from the rest of the previous eruption. All results are consistent.

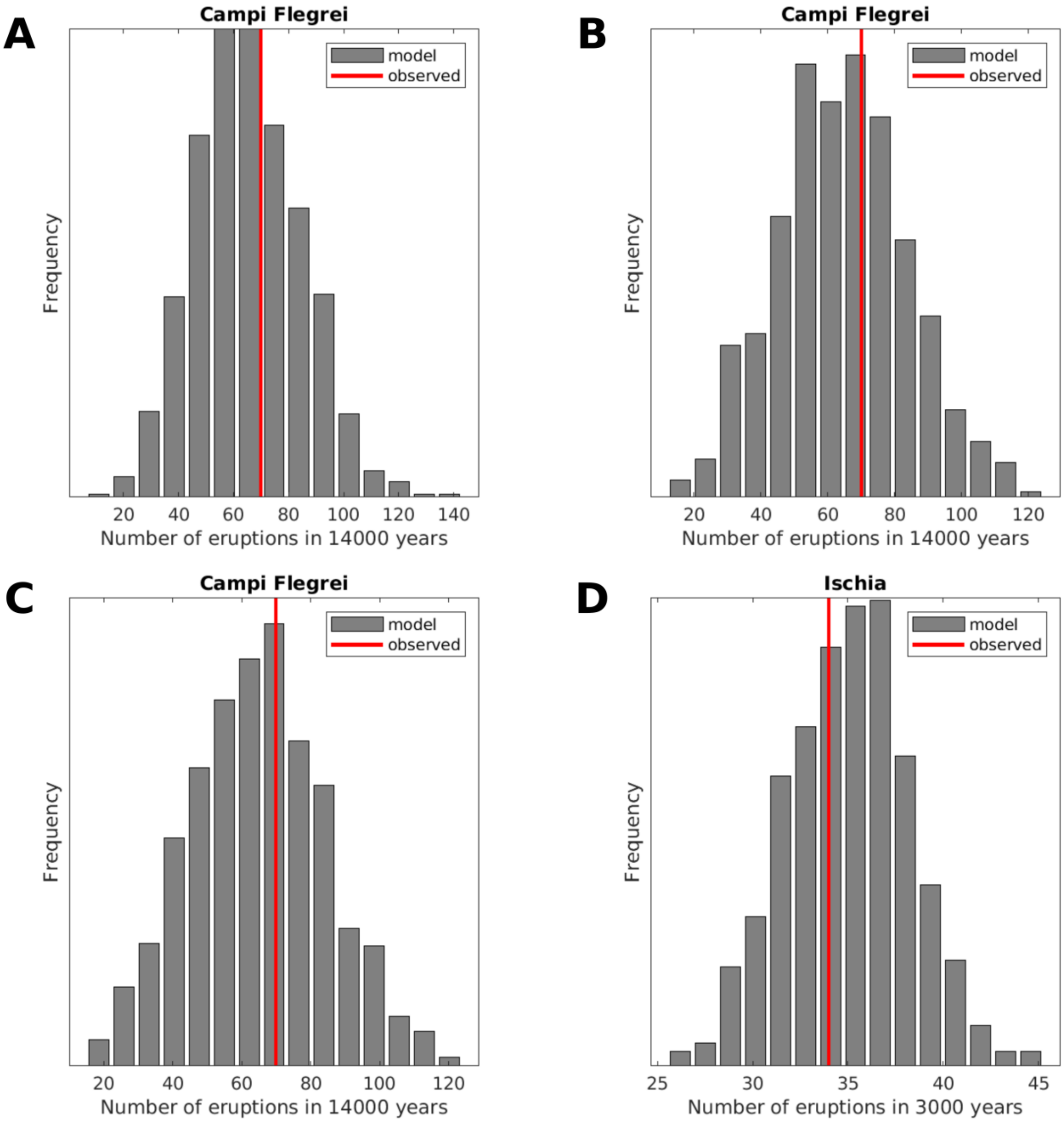

Fig. S3. Comparison with eruption counts. Same of Fig. 5, but with modified clusters. For Campi Flegrei, we consider 2 alternative hypotheses. In (A), we consider the Nisida eruption separated from Epoch 3, ending up with five clusters, the last two being isolated events (Nisida and Monte Nuovo). In (B), we consider the catalog considering only stratigraphic constraints. In (C), we consider an alternative catalog for Campi Flegrei, in which only stratigraphic constraints are used to set eruption dates, as suggested by the analysis of the eruption sequence. In (D), for Ischia, we consider 3 clusters, separating the Cretaio eruption (cluster with only 1 event) from the rest of the previous eruption. All results are consistent.

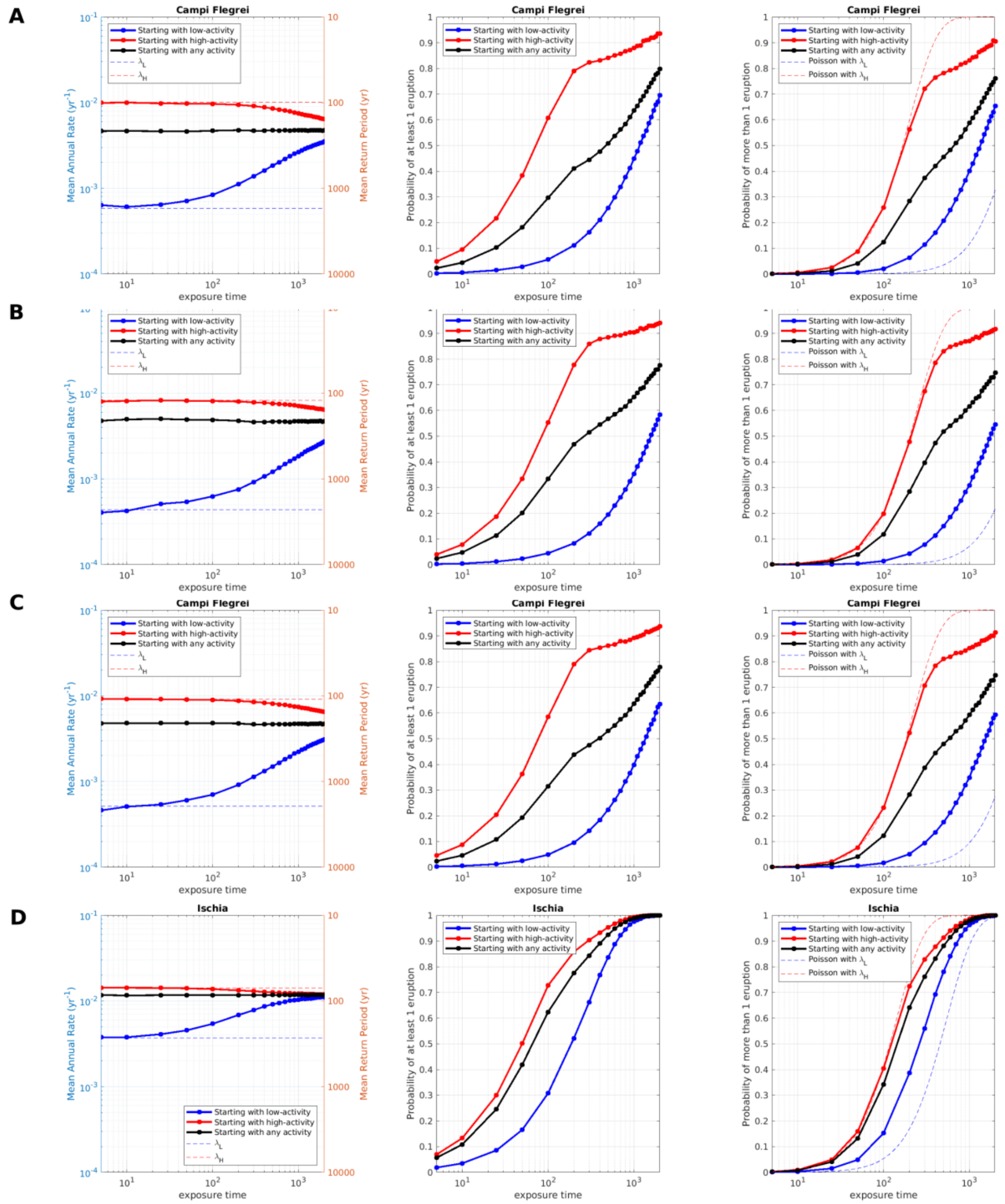

Fig. S4. Eruption probability. Same of Fig. 6, but with modified clusters. For Campi Flegrei, we consider 2 alternative hypotheses. In (A), we consider the Nisida eruption separated from Epoch 3, ending up with five clusters, the last two being isolated events (Nisida and Monte Nuovo). In (B), we consider only 3 clusters, merging Epochs I and II. In (C), we consider an alternative catalog for Campi Flegrei, in which only stratigraphic constraints are used to set eruption dates, as suggested by the analysis of the eruption sequence. In (D), for Ischia, we consider 3 clusters, separating the Cretaio eruption (cluster with only 1 event) from the rest of the previous eruption. All results are consistent.

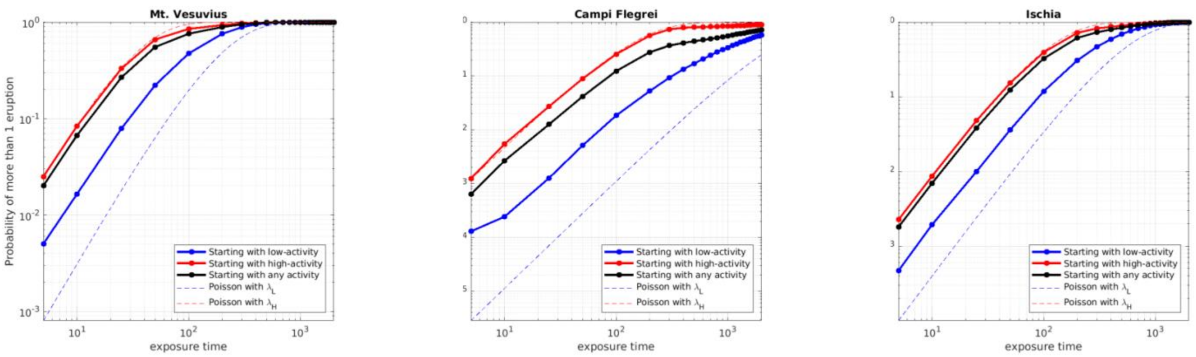

Fig. S5. Eruption probability. Same of Fig. 6C, but in logarithmic scale.

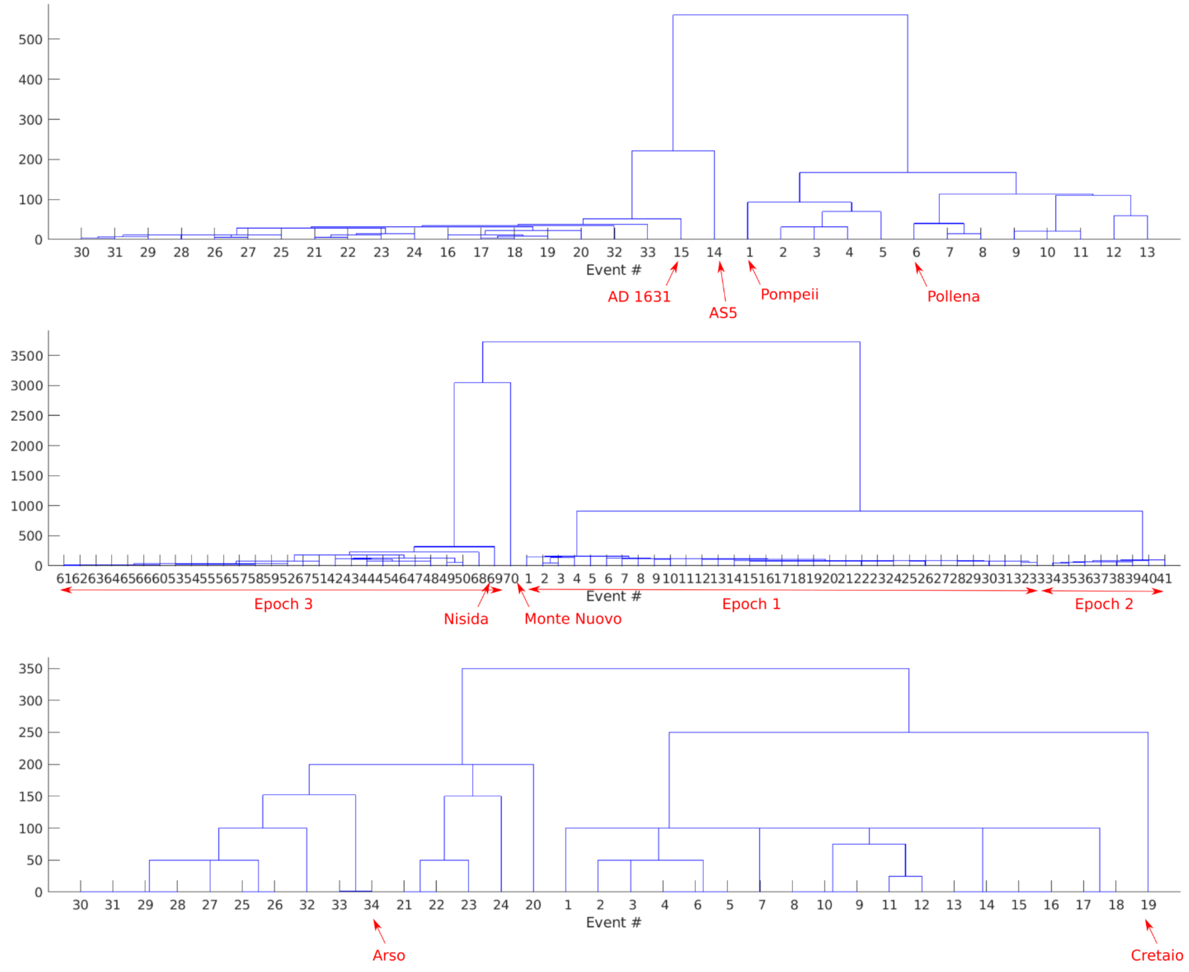

Fig. S6. Cluster Analysis. Dendrogram of a standard Hierarchical Cluster Analysis with Euclidean distances on available catalogs for Mt. Vesuvius (top), Campi Flegrei (middle), and Ischia (bottom). Numbers correspond to individual eruptions, as listed in Tables S1, S2 and S3.

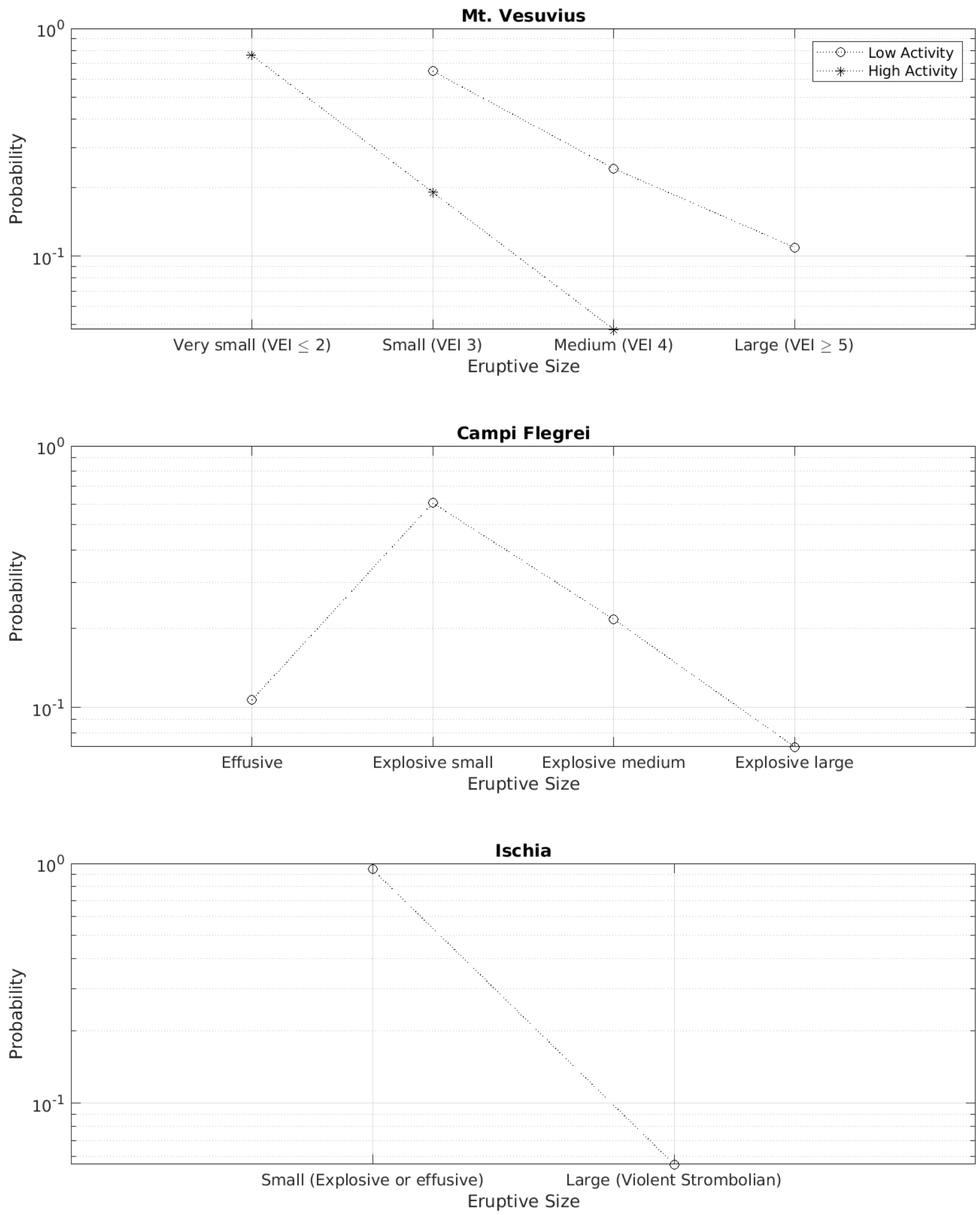

Fig. S7. Frequency size distributions. Frequency size distributions for Mt. Vesuvius (top), Campi Flegrei (middle), and Ischia (bottom). Distributions for low- and high-activity periods are assumed equal for both Campi Flegrei and Ischia.

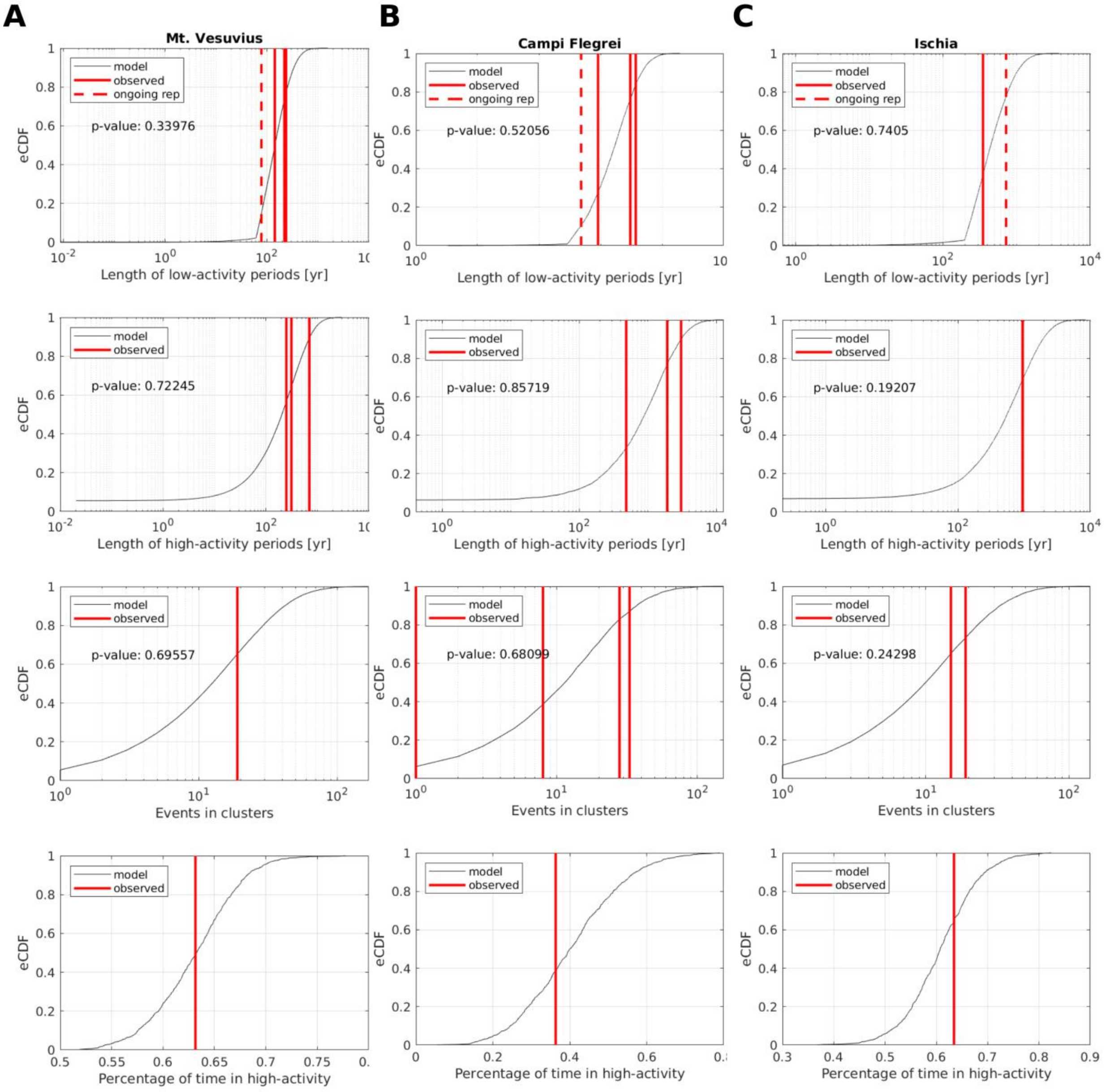

Fig. S8. Comparison with data (cumulative). As Fig. 4, but using the empirical cumulative distribution function, to better compare observations with percentiles. The p-values reported in several panels are relative to a Kolmogorov-Sminov one-sample test to compare observed and simulated distributions, reported also in Table 2.

Table S1. Mt. Vesuvius Catalog. Eruption catalog for Mt. Vesuvius.

#	Year AD	Name	Cluster
1	79	Pompeii	Pompeii sequence
2	172	Santa Maria Cycle	Pompeii sequence
3	203	Santa Maria Cycle	Pompeii sequence
4	235	Santa Maria Cycle	Pompeii sequence
5	305	Santa Maria Cycle	Pompeii sequence
6	472	Pollena	Pollena sequence
7	512	AS1	Pollena sequence
8	526	AS2	Pollena sequence
9	640	AS3 (PM3)	Pollena sequence
10	660	AS3 (PM4)	Pollena sequence
11	680	AS3 (PM5)	Pollena sequence
12	790	AS4	Pollena sequence
13	850	AS4b	Pollena sequence
14	1410	AS5	Isolated
15	1631	AD 1631 eruption	1631 sequence
16	1682	1682 cycle (1638-1682)	1631 sequence
17	1694	1694 cycle (1685-1694)	1631 sequence
18	1698	1698 cycle (1696-1698)	1631 sequence
19	1707	1707 cycle (1700-1707)	1631 sequence
20	1730	1723 and 1730 cycle (1712-1737)	1631 sequence
21	1761	1761 cycle (1742-1761)	1631 sequence
22	1767	1767 cycle (1764-1767)	1631 sequence
23	1779	1779 cycle (1770-1779)	1631 sequence
24	1794	1794 cycle (1783-1794)	1631 sequence
25	1822	1822 cycle (1799-1822)	1631 sequence
26	1834	1834 cycle (1825-1834)	1631 sequence
27	1839	1839 cycle (1835-1839)	1631 sequence
28	1850	1850 cycle (1841-1850)	1631 sequence
29	1861	1861 cycle (1854-1861)	1631 sequence
30	1868	1868 cycle (1864-1868)	1631 sequence
31	1872	1872 cycle (1870-1872)	1631 sequence
32	1906	1906 cycle (1874-1906)	1631 sequence
33	1944	1944 cycle (1913-1944)	1631 sequence

Table S2. Campi Flegrei catalog. Eruption catalog for Campi Flegrei. Dates are the mean of the epistemic uncertainty.

#	Year AD	Name	Cluster
1	-11600	Bellavista	Epoch 1
2	-11455	Mofete	Epoch 1
3	-11406	Gauro	Epoch 1
4	-11269	Santa Teresa	Epoch 1
5	-11106	La Pietra	Epoch 1
6	-10946	La Pigna 1	Epoch 1
7	-10821	La Pigna 2	Epoch 1
8	-10701	Torre Cappella	Epoch 1
9	-10585	Minopoli 1	Epoch 1
10	-10475	Paradiso	Epoch 1
11	-10360	Soccavo 1	Epoch 1
12	-10244	Gaiola	Epoch 1
13	-10139	Pomici Principali	Epoch 1
14	-10032	Paleo Pisani 1	Epoch 1
15	-9948	Paleo Pisani 2	Epoch 1
16	-9870	Soccavo 2	Epoch 1
17	-9792	Soccavo 3	Epoch 1
18	-9714	S4s3_1	Epoch 1
19	-9636	S4s3_2	Epoch 1
20	-9564	Soccavo 4	Epoch 1
21	-9483	Paleo San Martino	Epoch 1
22	-9404	Minopoli 2	Epoch 1
23	-9327	Soccavo 5	Epoch 1
24	-9247	Pisani 1	Epoch 1
25	-9165	Pisani 2	Epoch 1
26	-9085	Fondo Riccio	Epoch 1
27	-9002	Concola	Epoch 1
28	-8920	Montagna Spaccata	Epoch 1
29	-8842	Pignatiello 1	Epoch 1
30	-8761	Pisani 3	Epoch 1
31	-8682	Casale	Epoch 1
32	-8609	Bacoli	Epoch 1
33	-8550	Porto Miseno	Epoch 1
34	-7640	Baia	Epoch 2

35	-7591	Fondi di Baia	Epoch 2
36	-7530	Sartania 1	Epoch 2
37	-7465	Monte Spina lava dome	Epoch 2
38	-7402	Costa San domenico	Epoch 2
39	-7339	Pigna San Nicola	Epoch 2
40	-7249	Sartania 2	Epoch 2
41	-7157	San Martino	Epoch 2
42	-3424	Agnano 1	Epoch 3
43	-3311	Agnano 2	Epoch 3
44	-3202	Averno 1	Epoch 3
45	-3125	Agnano 3	Epoch 3
46	-3052	Cigliano	Epoch 3
47	-2976	Pigniatiello 2	Epoch 3
48	-2901	Capo Miseno	Epoch 3
49	-2768	Monte Sant’Angelo	Epoch 3
50	-2713	Paleoastroni 1	Epoch 3
51	-2530	Paleoastroni 2	Epoch 3
52	-2449	Agnano Monte Spina	Epoch 3
53	-2409	St. Maria delle Grazie	Epoch 3
54	-2379	Olibano lava dome	Epoch 3
55	-2350	Paleoastroni 3	Epoch 3
56	-2322	Solfatara lava dome	Epoch 3
57	-2292	Olibano tephra	Epoch 3
58	-2263	Accademia lava dome	Epoch 3
59	-2230	Solfatara	Epoch 3
60	-2195	Averno 2	Epoch 3
61	-2183	Astroni 1	Epoch 3
62	-2173	Astroni 2	Epoch 3
63	-2163	Astroni 3	Epoch 3
64	-2152	Astroni 4	Epoch 3
65	-2142	Astroni 5	Epoch 3
66	-2132	Astroni 6	Epoch 3
67	-2052	Astroni 7	Epoch 3
68	-1825	Fossa Lupara	Epoch 3
69	-1507	Nisida	Epoch 3
70	1538	Monte Nuovo	Isolated

Table S3. Ischia catalog. Eruption catalog for Ischia. Uncertainty on dating is not considered.

#	Year AD	Name	Cluster
1	-951	Cannavale Tephra	Cretaio cluster
2	-851	Cantariello Lavas	Cretaio cluster
3	-801	Punta La Scrofa Tephra	Cretaio cluster
4	-751	Marecoppo Tephra	Cretaio cluster
5	-751	San Ciro Lavas	Cretaio cluster
6	-751	Chiarito Tephra	Cretaio cluster
7	-651	Monte Toppo Lavas	Cretaio cluster
8	-551	Montagnone I Lavas	Cretaio cluster
9	-551	San Pietro Lavas	Cretaio cluster
10	-551	Molara Tephra	Cretaio cluster
11	-476	Ischia Porto Tephra	Cretaio cluster
12	-451	Vateliero Tephra	Cretaio cluster
13	-351	Cava Bianca Tephra	Cretaio cluster
14	-351	Posta Lubrano Lavas	Cretaio cluster
15	-351	Cafieri Tephra	Cretaio cluster
16	-351	Cafieri Lavas	Cretaio cluster
17	-251	Posta Lubrano Tephra	Cretaio cluster
18	-251	Cava Nocelle Tephra	Cretaio cluster
19	-1	Cretaio Tephra	Cretaio cluster
20	349	Arcamone Sacchetta Lavas	Arso cluster
21	549	Cava Buceto Tephra	Arso cluster
22	549	Bosco dei Conti Tephra	Arso cluster
23	599	Bosco Maddalena Lavas	Arso cluster
24	749	Villammare Tephra	Arso cluster
25	949	Tabor Lavas	Arso cluster
26	949	Fondo d'Oglio Tephra	Arso cluster
27	999	Bosco Maddalena Tephra	Arso cluster
28	1049	Fiaiano Tephra	Arso cluster
29	1049	Punta La Scrofa Lavas	Arso cluster
30	1049	Montagnone-Maschiata	Arso cluster
31	1049	Rotaro Lavas	Arso cluster
32	1149	Pietra Vono	Arso cluster
33	1301	Fondo Bosso Tephra	Arso cluster
34	1302	Arso Lavas and Tephra	Arso cluster
